# Dimeric gold nanoparticles enable multiplexed labeling in cryoelectron tomography

**DOI:** 10.1073/pnas.2524034122

**Published:** 2025-11-24

**Authors:** Hoyoung Kim, Cathy J. Spangler, Aya Matsui, Johannes Elferich, Junhoe Kim, Alex Roseborough, May Nyman, Tanja M. Lahtinen, Eric Gouaux

**Affiliations:** ^a^Vollum Institute, Oregon Health and Science University, Portland, OR 97239; ^b^HHMI, Oregon Health and Science University, Portland, OR 97239; ^c^RNA Therapeutics Institute, University of Massachusetts Chan Medical School, Worcester, MA 01605; ^d^HHMI, University of Massachusetts Chan Medical School, Worcester, MA 01605; ^e^Department of Chemistry, Oregon State University, Corvallis, OR 97331; ^f^Department of Chemistry, Nanoscience Center, University of Jyväskylä, Jyväskylä Fl-40014, Finland

**Keywords:** gold nanoparticles, cryoelectron tomography, NMDA receptors, multiplexed labeling, synaptic cleft

## Abstract

Cryoelectron tomography (cryo-ET) enables three-dimensional visualization of molecular structures within tissue and intact cells, providing a powerful tool for studying the spatial organization of biological components at nanometer resolution. Realizing this potential, particularly for submegadalton complexes, is facilitated by fiducial-based labeling. Gold nanoparticles (AuNPs) have emerged as powerful electron-dense labels for cryo-ET, but multiplexing using only conventional monomeric AuNPs is challenging, limiting their application in multitarget studies. Here, we describe functionalized dimeric AuNPs with a precisely defined size range applied to bioimaging, enabling multiplexed labeling by allowing reliable discrimination between monomeric and dimeric AuNPs, thereby supporting identification of distinct molecular targets within the same cryotomogram. Each dimer consists of two covalently linked gold particles of approximately 2.6 nm diameter separated by a defined spacing of about 1.4 nm, with high structural homogeneity validated by small-angle X-ray scattering (SAXS) and electron microscopy. The anti-GluN1 Fab, which targets the *N*-methyl-D-aspartate receptor (NMDAR), was site specifically conjugated to a dimeric AuNP. A deep learning classifier enabled reliable discrimination between monomeric and dimeric AuNPs in tomograms. We confirmed that the dimeric AuNP–Fab conjugates bind robustly to recombinant GluN1/GluN2A receptors, validating their use for structural labeling. In situ cryo-ET of brain tissue further confirms that the dimeric labels reach NMDARs within the glutamatergic synaptic cleft. Combined with monomeric AuNPs, this dimeric AuNP platform establishes a generalizable approach for distinguishable labels optimized for cryo-ET. The compact size and structural uniformity of monomeric and dimeric AuNPs make them ideally suited for nanoscale molecular mapping in crowded cellular environments.

## Significance

Understanding how proteins are organized within the crowded and geometrically constrained environment of tissues and cells is a major goal of structural biology. Cryoelectron tomography (cryo-ET) offers a powerful means to visualize molecular architecture in situ, but its full potential has been limited by the lack of multiplexed labeling tools. Here, we introduce a compact and structurally defined dimeric gold nanoparticle (AuNP) labeling strategy that enables simultaneous discrimination of multiple molecular targets within a single tomogram. By combining geometrically encoded, Fab-based specificity and deep learning-driven classification, this approach augments the potential of monomeric AuNPs. The resulting platform expands the toolkit for nanoscale molecular mapping and opens opportunities for high-resolution structural analysis in intact tissues.

Since the pioneering immunogold-labeling studies of the early 1970s, when Faulk and Taylor (1) first demonstrated the use of colloidal gold for antigen localization in electron microscopy (EM) and Horisberger et al. (2) extended the approach to sub-30 nanometer (nm) particles, gold particles have proven to be versatile labels for EM. Cryo-electron tomography (cryo-ET) is now essential in cellular and structural biology, enabling three-dimensional determination of biomolecular structures under near-native conditions (3, 4). More recently, gold nanoparticles (AuNPs) have emerged as powerful tools for site-specific labeling and visualization of biomolecules across cryo-EM and cryo-ET platforms (5). Their intrinsic electron-dense nature, tunable surface chemistry, and sub-5 nm dimensions make them ideal for tagging macromolecules in crowded cellular environments (6). Furthermore, there is growing interest in using more than one distinct label within a single sample to dissect multiple biological relationships simultaneously (7). AuNPs are particularly well suited for this purpose, as they can be prepared in a range of sizes beyond the commonly used 2 nm particles (8). AuNPs of 5 nm or even 10 nm diameter can be deployed as additional labels (9). In addition to AuNPs, alternative high-contrast markers such as apo or iron-loaded ferritin complexes can also be used for multiplexed cryo-ET labeling (10).

Larger fiducial labels, however, introduce important limitations (11). Particles exceeding 2 nm in diameter such as 5 nm or 10 nm AuNPs and 14 nm ferritin complexes can encounter or introduce steric clashes in confined cellular compartments, thereby reducing labeling efficiency and distorting spatial localization (12). In highly crowded environments, such as the synaptic cleft, which is ~28 nm in width (13), large fiducial markers may prevent access to target sites altogether. Moreover, their bulk may perturb native protein conformation, localization, or interaction. As a result, although larger AuNPs and ferritin enable multiplexed labeling by providing distinguishable contrast signatures, their utility is limited by both physical inaccessibility and the potential for structural interference (14). This constraint is particularly critical at the synapse, where receptors, scaffolding proteins, and signaling complexes are densely packed within a nanometer-scale volume (15). Understanding the spatial organization of key components such as α-amino-3-hydroxy-5-methyl-4-isoxazolepropionic acid receptors (AMPARs), *N*-methyl-D-aspartate glutamate receptors (NMDARs), and voltage-gated calcium channels requires labeling strategies that are both sufficiently compact to access the narrow synaptic cleft and capable of providing multiple distinguishable markers for precise molecular localization (16, 17).

To address this challenge, we developed a labeling strategy based on highly homogeneous dimeric AuNPs composed of two covalently linked ~2.6 nm particles (18). This configuration maintains the uniform size distribution characteristic of monomeric AuNPs and, despite being larger than a single AuNP, remains sufficiently compact to access confined spaces such as the synaptic cleft. Moreover, the molecularly fixed nanoscale gap between the two AuNPs within a dimer enables the reliable identification of the labels and the determination of their orientation. We implemented this approach by constructing covalently linked, dimeric AuNPs conjugated to engineered Fab fragments targeting the GluN1 NMDAR subunit. This dimeric AuNP configuration provides several advantages for in situ labeling. It retains the intrinsic benefits of AuNPs, as it employs the same stable and well-characterized 2.6 nm AuNPs commonly used in cryo-ET studies. Because this synthesis method produces a single, well-defined type of AuNP, their behavior in biological environments remains consistent, thereby supporting reproducibility of labeling results and minimizing uncertainties about nanoparticle-induced effects on biomolecules (18). Importantly, by simply adding one additional 2.6 nm particle (r = 1.3 nm; V ≈ 9.2 nm^3^ per particle), the combined volume (~18.4 nm^3^) remains substantially smaller than that of a single 5 nm (r = 2.5 nm; V ≈ 65.4 nm^3^) or 10 nm (r = 5 nm; V ≈ 523.6 nm^3^) AuNP, making this dual-particle strategy more compatible with confined environments such as the synaptic cleft.

Together, these results establish a versatile and generalizable framework for dual-site labeling in structural biology, demonstrating that the distinct geometries of monomeric versus dimeric AuNPs enable precise dual-site labeling of molecular targets with high specificity and spatial resolution.

## Results

### Monomer AuNP Synthesis, AuNP Dimer Synthesis, and Fab Conjugation.

To prepare Fab-conjugated dimeric AuNPs we followed three main steps: i) synthesis of monomeric AuNPs, ii) assembly and purification of dimeric AuNPs, and iii) conjugation of Fab fragments to the dimeric AuNPs followed by PEGylation (*SI Appendix*, Fig. S1). First, monomeric AuNPs were synthesized and then cross-linked with biphenyl-4,4′-dithiol (BPDT) to generate multimeric assemblies (18). These assemblies were resolved by native PAGE to isolate the dimeric fraction. Next, Fab fragments bearing an engineered free cysteine residue were conjugated to the purified dimeric AuNPs via a Murray place-exchange reaction (19). Finally, to reduce surface reactivity and enhance colloidal stability, the Fab–AuNP conjugates were PEGylated. To enhance the overall yield and homogeneity of the desired Fab-dimeric AuNP conjugate, we optimized all three steps of the synthetic pathway. With the workflow overview now complete, we turn to a detailed, step-by-step description of each method beginning with the synthesis of dimeric AuNPs.

### Synthesis of Dimeric AuNPs.

**Fig. 1.**
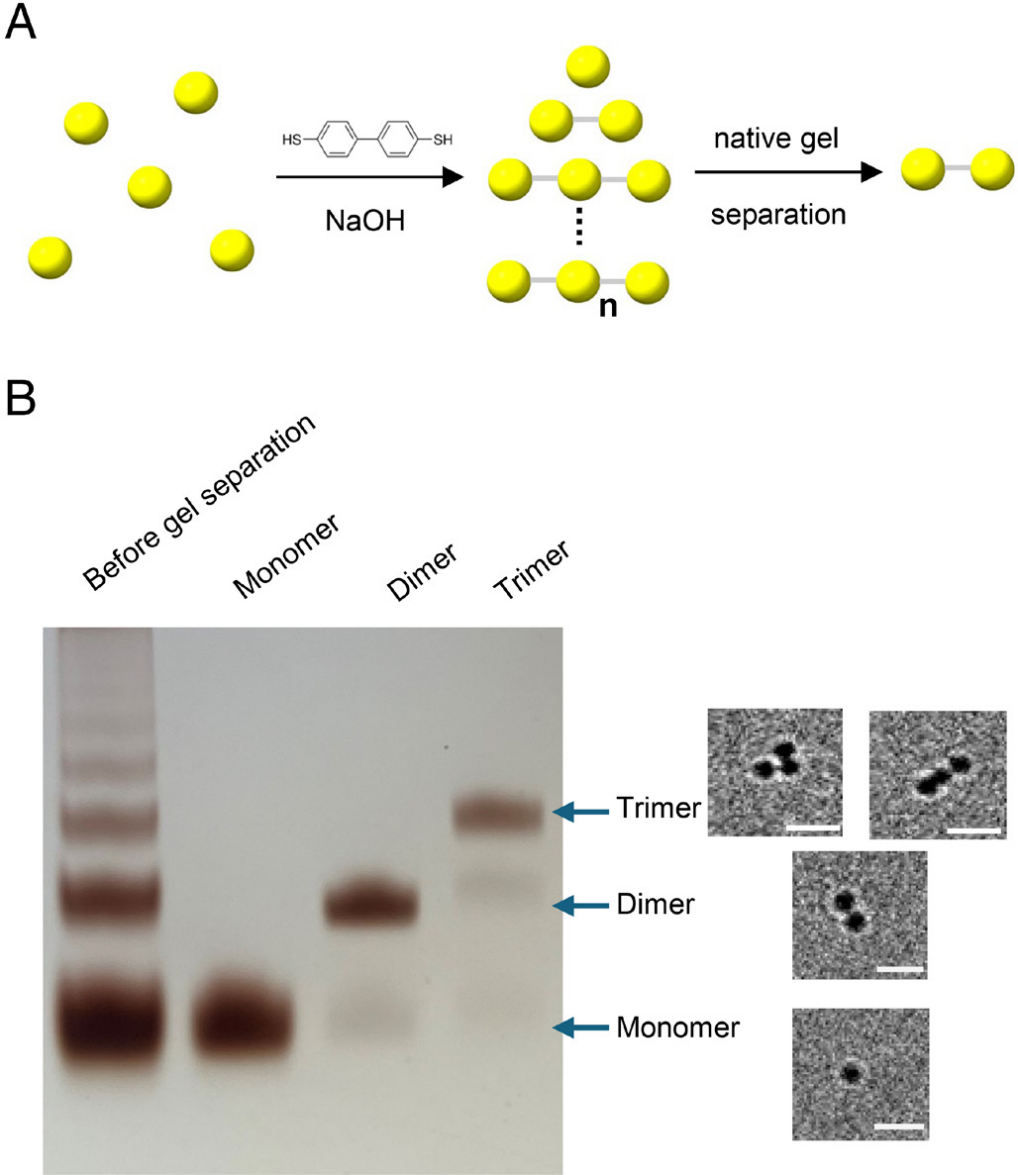
Synthesis, analysis of the coupling reaction mixture, and isolation of defined AuNP. (*A*) Schematic of the BPDT-mediated strategy for generating dimeric AuNPs. (*B*) Native gel electrophoresis of multimeric AuNPs, showing the separation of different AuNP multimers, with representative TEM images of the material eluted from each band. The white scale bar in the micrographs represents 10 nm.

To generate dimeric AuNPs via BPDT cross-linking, we first synthesized ~2.6 nm monomeric AuNPs coated with 3-mercaptobenzoic acid (3-MBA) (20) and then reacted them with BPDT under alkaline conditions (18). This yields a mixture of multimeric AuNPs which can be resolved by native PAGE (Fig. 1*A*). Before purification, multiple discrete bands corresponding to different oligomeric species are visible on the gel. The bands corresponding to monomer, dimer, and trimer were excised and eluted from the gel, then rerun on native PAGE to confirm isolation of the desired oligomeric species. Although the major species were well separated, minor carry-over of adjacent oligomeric states is evident in each fraction. Cryo-EM analysis of the eluted samples shows that the monomer, dimer, and trimer fractions predominantly contain particles bearing one, two, or three AuNPs, respectively, and gel band intensity analysis (21) further indicated that the dimer and trimer fractions had purities of approximately 94% and 83%, respectively. While trimers could theoretically serve as labels, the presence of both triangular and linear geometries makes them unsuitable for uniform labeling (Fig. 1*B*). The apparently linear configuration may in fact represent a side view of the triangular arrangement, and we note this possible ambiguity in interpreting trimer structures.

### Structural Characterization of Monomeric and Dimeric AuNPs.

**Fig. 2.**
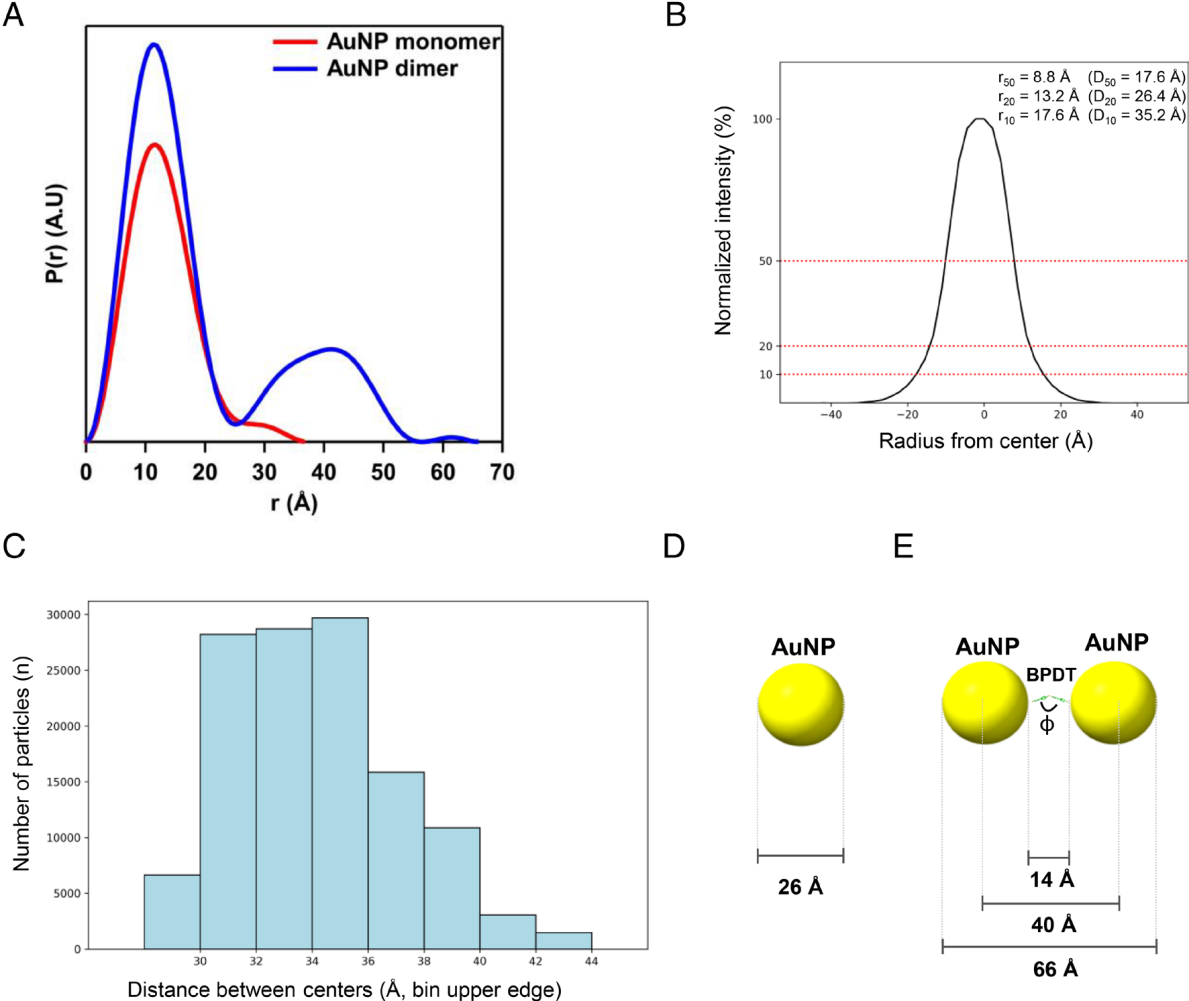
Analysis of monomeric and dimeric AuNPs by SAXS and single-particle cryo-EM. (*A*) Pair distance distribution functions (PDDFs) derived from SAXS profiles of aqueous monomeric and dimeric AuNP solutions (1 to 2 mg/mL). (*B*) Radial intensity profiles for monomeric AuNPs (n = 79,897 particles) derived from cryo-EM 2D class averages. 2D class averages were aligned, recentered on the particle midpoint, and mean intensity was plotted as a function of radial distance. Dotted lines mark the cutoff radii r_50_, r_20_, and r_10_ (corresponding to 50, 20, and 10% intensity levels), with negative radii mirrored symmetrically about the origin. (*C*) Binned distribution of dimeric AuNP separations (n = 124,511). Twenty cryo-EM 2D class averages with clearly separated dimers were aligned, and each centroid was located. The center-to-center separation measured for each class was aggregated into 2 Å bins from 26 to 46 Å. Bars show the particle count per bin; x-axis labels indicate the *Upper* edge of each bin (bars are *Left*-aligned to the *Lower* edge). Schematic model of the monomeric (*D*) and dimeric AuNP (*E*), with the measured interparticle distance indicated.

Small-angle X-ray scattering (SAXS) measurements were conducted on unPEGylated, 3-MBA-coated monomeric and dimeric AuNPs to assess particle size and shape in bulk solutions. The scattering curve of monomeric AuNPs features a typical Guinier elbow between q ~ 0.07 to 0.3 Å, characteristic of a solution of monodisperse spherical particles and a radius of gyration (R_g_, root mean squared electron distance from center of mass of particle) of 9.96 (±0.01) Å, as determined by pair distance distribution function (PDDF), using the method of Moore (22). PDDF (Fig. 2*A* and *SI Appendix*, Fig. S2*A*) is a probability distribution map of scattering vectors through the particles, where characteristic profiles of the PDDF describe the shape of the scattering particle. The PDDF for the AuNP monomers suggests spherical particles of ~25 Å diameter for the gold core (23). In contrast, the dimeric AuNPs show increased intensity at I_0_ (forward-scattering intensity) (q ≈ 0.017 Å^−1^) and a new shoulder at q ≈ 0.2 Å^−1^; PDDF analysis (Fig. 2*A* and *SI Appendix*, Fig. S2*B*) gives R_g_ = 16.50 ± 0.01 Å and a maximum particle dimension (D_max_) of ~65 Å. The two peak profiles, with the second of lower intensity than the first, are consistent with a two-particle dimer, where the first peak maximum I(r) (12 Å) is the radius of the monomer particle (24). Finally, the third small peak in the PDDF corresponds to a low-electron-density surfactant shell surrounding the gold core (25), consistent with the 3-MBA coating.

To assess the diameters of monomeric and dimeric AuNPs, cryo-EM micrographs were subjected to 2D classification (*SI Appendix*, Fig. S3*A*). Monomeric AuNP class averages were centered and radial intensity profiles extracted; the 50%, 20%, and 10% cutoff distances correspond to diameters of ~17.6 Å, 26.4 Å, and 35.2 Å, respectively (Fig. 2*B*). Dimeric particle images were subjected to reference-free 2D classification into 20 classes, a number empirically determined to balance class homogeneity and particle count. For each class average, the two intensity-weighted centroids were connected by a straight line, and intensity was sampled along this intercentroid axis from -50 Å to +50 Å around the midpoint (0 Å). Center-to-center separations averaged 34.8 ± 3.6 Å (mean ± SD; *SI Appendix*, Fig. S3*B*). However, because particle counts decrease sharply beyond ~40 Å, we adopt 40 Å as the representative center-to-center separation (Fig. 2*C*). Based on SAXS and 2D cryo-EM analyses, the 20% intensity cutoff radius (r_20_ = 13.2 Å, D_20_ = 26.4 Å) was taken as the monomeric AuNP diameter (Fig. 2*D*). Assuming a dimeric separation of 40 Å and monomer diameter of 26.4 Å, the interparticle gap is ~13.6 Å, suggesting that two BPDT linkers in series could bridge this distance. The intramolecular S-S distance in BPDT spans ~7.5 Å (φ = 90°) to ~10.6 Å (φ = 0°) (26). Thus, a single BPDT cannot bridge 13.6 Å, but two BPDTs in series, by adopting intermediate torsional conformations, can readily produce the observed separation. The end-to-end distance of ~66 Å is consistent with the SAXS data (Fig. 2*E* and *SI Appendix*, Fig. S2).

### Design and Cryo-EM Validation of Cysteine-Engineered 5F11 Fab for AuNP Conjugation.

The 5F11 Fab binds the GluN1 subunit of the NMDAR and engages both the R1 and R2 lobes of its amino-terminal domain (ATD) (27). Cryo-EM reconstruction of the 5F11 Fab–NMDA complex at ~11 Å resolution confirmed binding to the GluN1 ATD, indicating that C-terminal cysteine conjugation to AuNPs preserves antigen recognition (*SI Appendix*, Fig. S4 *A*−*C*). To enable AuNP conjugation, the anti-GluN1 5F11 Fab was engineered with cysteine mutations at heavy chain position C229 and light chain position C222, allowing stable and site-specific conjugation (*SI Appendix*, Fig. S4*D*). These residues are positioned on the extended, unstructured C termini of the respective chains to minimize disruption to Fab folding or antigen binding.

### Conjugation and PEGylation of 5F11 Fab-Dimeric AuNP.

Cysteine-engineered 5F11 Fabs were expressed, purified, and conjugated to dimeric AuNPs. To optimize the AuNP conjugation reaction, we performed a molar-ratio titration (*SI Appendix*, Fig. S5*A*). Unlike monomeric AuNPs, which provide only one thiol-reactive spherical surface for Fab conjugation, dimeric AuNPs comprise two linked ~2.6 nm particles, each with its own thiol-reactive spherical surface. Our goal was to produce a 1:1 Fab:dimeric AuNP conjugate, with one Fab molecule bound to each dimeric AuNP. We tested various Fab to dimeric AuNP molar ratios and found that a 1:2 (Fab:AuNP) reagent ratio maximized the yield of the 1:1 Fab:dimeric AuNP conjugate; these conditions were selected for scale-up. After conjugating 5F11 Fab with dimeric AuNP, the conjugates were purified via native gel electrophoresis and elution, yielding highly pure 5F11 Fab-dimeric AuNPs (*SI Appendix*, Fig. S5*B*).

**Fig. 3.**
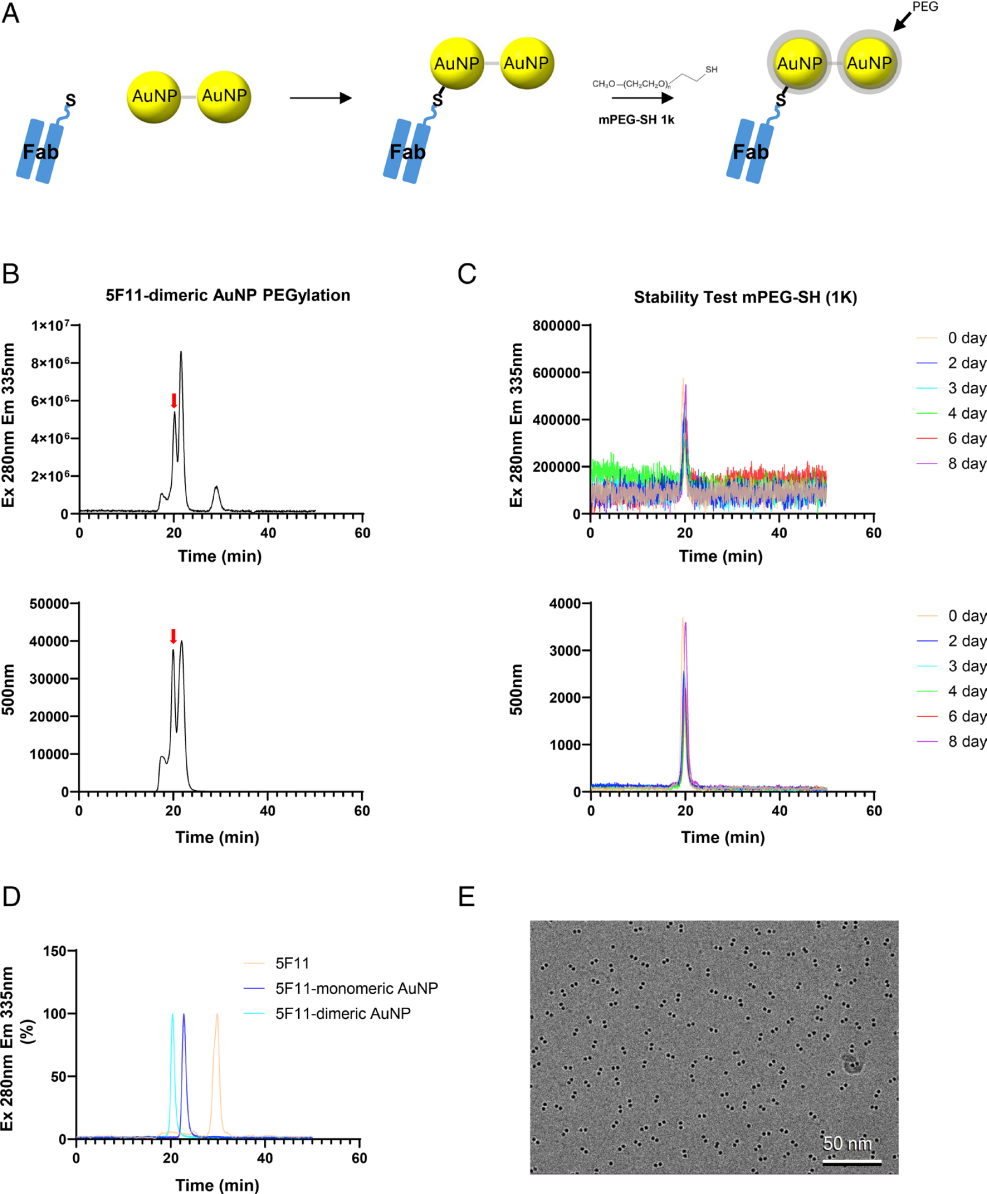
PEGylation and stability analysis of 5F11-dimeric AuNP conjugates. (*A*) Schematic of conjugation strategy for covalent linkage and PEGylation of dimeric AuNP to Fab. (*B*) FSEC analysis showing the extent of PEGylation at 1K PEG for 5F11-dimeric AuNP. (The red arrow indicates the 5F11-dimeric AuNP.) (*C*) FSEC-based stability test of 5F11-dimeric AuNP-1K PEG over time at 4 °C. (*D*) Comparison of 5F11 Fab conjugation efficiency among monomeric AuNP, dimeric AuNP, and free Fab using FSEC analysis. (*E*) Cryo-EM micrograph of 5F11 Fab-dimeric AuNP-1K PEG, confirming its structural integrity.

To prepare tomography-compatible dimeric AuNP labels and enhance their stability while minimizing nonspecific binding, 5F11-conjugated dimers were PEGylated (Fig. 3*A*) (28). PEGylation was performed using methoxy polyethylene glycol thiol (mPEG-SH), which contains a thiol group for AuNP binding. Three different molecular weights of mPEG-SH (350, 550, and 1 K) were tested (*SI Appendix*, Fig. S6*A*). The reaction was conducted at five different concentrations (0.5, 1, 2, 3, and 5 mM), followed by size exclusion chromatography (SEC) for separation (*SI Appendix*, Fig. S6 *B*−*D*). PEGylation resulted in multiple fractions, indicating the formation of various PEGylated species. For mPEG-SH (350), the yield of the target 5F11 Fab-dimeric AuNP was minimal, so further experiments with this PEG size were discontinued. Instead, only mPEG-SH (550) and mPEG-SH (1K) were employed in subsequent PEGylation reactions. We performed PEGylation at a PEG concentration of 0.5 mM and selectively collected the target fraction for further analysis (Fig. 3*B*).

To assess the long-term stability of the PEGylated dimeric AuNPs, samples were stored at 4 °C for one week. The mPEG-SH (550) sample showed instability, aggregating into a void peak after four days (*SI Appendix*, Fig. S7). In contrast, the mPEG-SH (1K) sample remained stable even after one week, demonstrating its superior stabilizing effect on 5F11 Fab-dimeric AuNP (Fig. 3*C*). The formation of 5F11 Fab-dimeric AuNP was confirmed through fluorescence size-exclusion chromatography (FSEC) (29), where differences in the sizes of 5F11 Fab, 5F11 Fab-monomeric AuNP, and 5F11 Fab-dimeric AuNP were observed (Fig. 3*D*). Additionally, the formation of AuNP dimers was validated through cryo-EM analysis of 5F11 Fab-dimeric AuNP (Fig. 3*E*), and combined analysis of FSEC and cryo-EM data indicates that dimeric AuNP–Fab conjugates account for over 95% of the total population.

### Identification of Monomeric Versus Dimeric AuNPs in Tomograms.

**Fig. 4.**
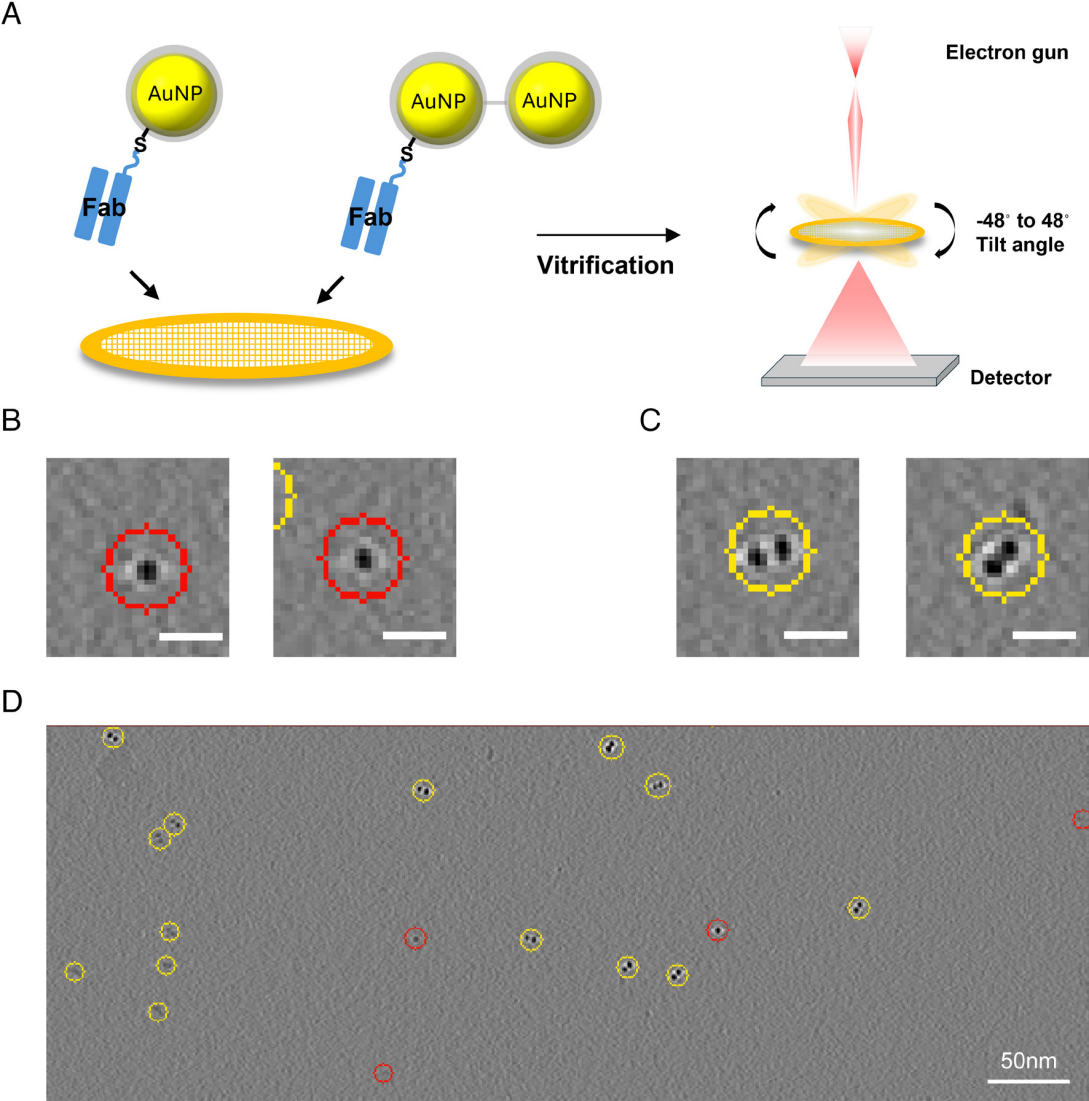
DeepETPicker-based analysis for distinguishing monomeric and dimeric AuNP in cryo-ET tilt series. (*A*) Schematic of the cryo-ET workflow: preparation of grids with 5F11-monomeric and dimeric AuNPs, followed by tilt-series collection (−48° to +48°). (*B*) Monomeric and (*C*) dimeric AuNPs were manually picked from tilt series data and categorized as Class 1 (red) and Class 2 (yellow), respectively. These manually curated datasets were used to train the DeepETPicker model. (*D*) Representative picking results obtained by applying the trained model to new tilt series data. The voxel size for each tomogram is 10 Å. White scale bars in (*B* and C) represent 10 nm.

To assess whether monomeric and dimeric AuNPs could be distinguished in tomograms, PEGylated 5F11-conjugated monomeric and dimeric AuNPs were mixed at a 1:2 molar ratio, applied to cryo-EM grids, plunge-frozen, and dose-symmetric tilt series were collected (Fig. 4*A*). Importantly, these tomograms were acquired from blotted grids, producing ice thickness more akin to single-particle analysis (SPA) than a typical tomogram experiment and yielding higher signal-to-noise. This thin-ice, high-SNR setup was chosen to give our machine-learning classifier the best chance to distinguish monomeric from dimeric AuNPs. Reconstructions at a 10 Å voxel size clearly resolved monomeric and dimeric particles. We then examined the effectiveness of the deep-learning picker DeepETPicker (30) to reliably pick monomeric or dimeric AuNPs. To do this, we manually picked monomeric particles (Class 1; Fig. 4*B*) and dimeric particles (Class 2; Fig. 4*C*) to train and refine the algorithm through two rounds of interface adjustment and manual correction. In the first round, some monomeric and dimeric picks were incorrect, but after correcting these mispicks and rerunning training, DeepETPicker achieved over 95% classification accuracy. The final DeepETPicker output accurately classified monomeric and dimeric AuNPs (Fig. 4*D*). Movie S1, generated in IMOD, shows representative tomogram slices overlaid with DeepETPicker classification masks, demonstrating how the refined model reliably discriminates monomeric (Class 1, red) and dimeric (Class 2, yellow) AuNPs after the second training round.

### 5F11 Fab-Dimeric AuNP Binds to Recombinant NMDARs.

**Fig. 5.**
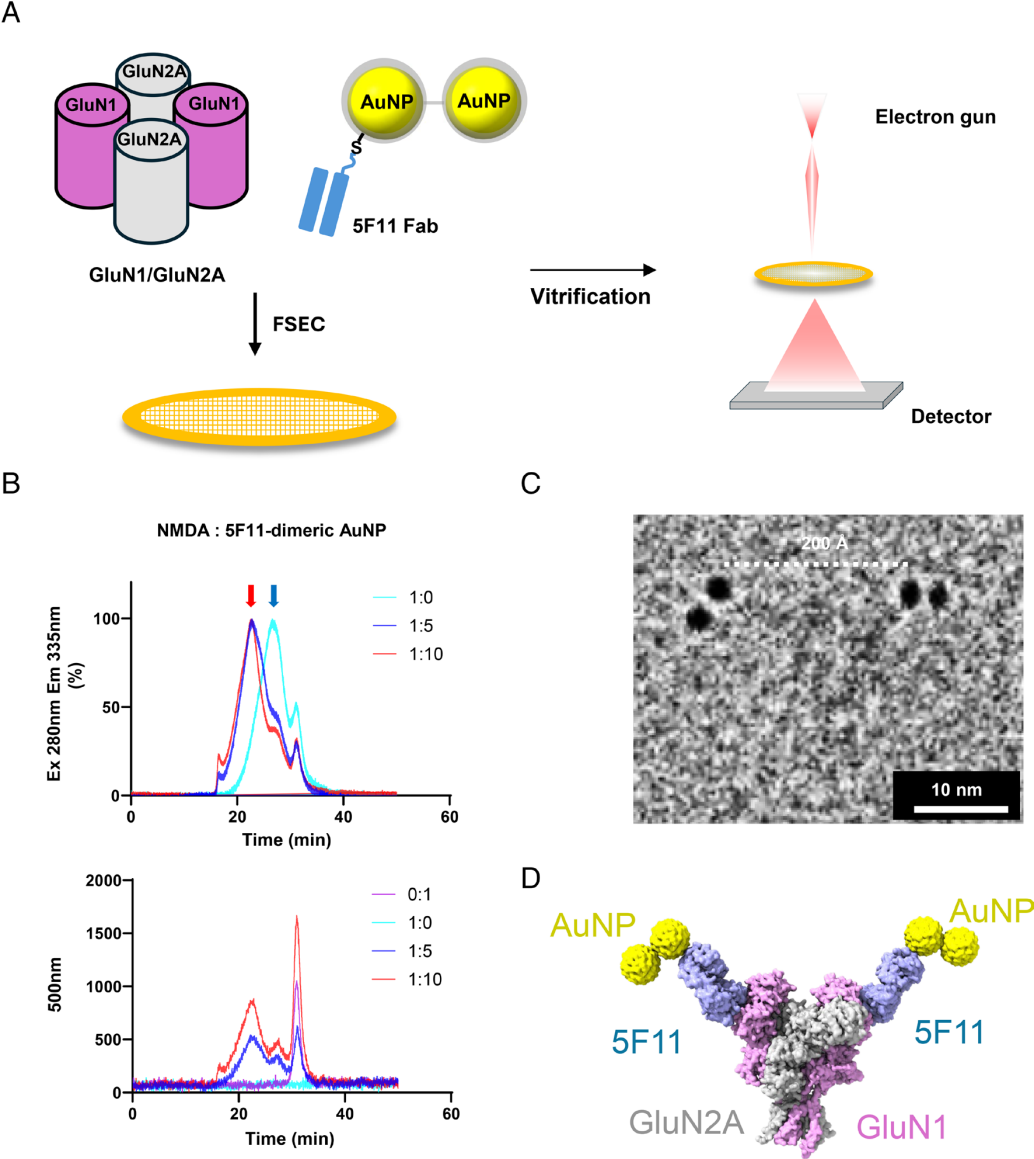
Binding of 5F11-dimeric AuNP to the NMDAR. (*A*) Schematic of the cryo-EM workflow: preparation of grids containing the NMDAR with 5F11-dimeric AuNPs, followed by cryo-EM data collection. (*B*) FSEC analysis of recombinant GluN1/GluN2A mixed with PEGylated 5F11-dimeric AuNP at molar ratios of 1:0, 1:5, and 1:10 (GluN1/2A:PEG5F11-dimeric AuNP). Tryptophan fluorescence (Ex 280 nm, Em 335 nm) exhibits a clear peak shift upon complex formation (red arrow: complex; blue arrow: unbound receptor), and the AuNP absorbance at 500 nm shows an increased peak height at the same elution volume. (*C*) Representative cryo-EM micrograph confirming binding of dimeric AuNP to the GluN1/GluN2A receptor. (*D*) Structural model of the GluN1/GluN2A 5F11-dimeric AuNP complex.

To confirm that the engineered 5F11 Fab-dimeric AuNP complex binds to purified recombinant GluN1/GluN2A receptors, we incubated the NMDARs with PEGylated 5F11 monomeric and dimeric AuNPs at receptor:AuNP molar ratios of 1:0, 1:5, and 1:10. The mixtures were applied to cryo-EM grids, and micrographs were collected (Fig. 5*A*). FSEC monitoring of tryptophan fluorescence (excitation 280 nm, emission 335 nm) and AuNP absorbance (500 nm) revealed binding induced peak shifts of the receptor complex in proportion to the AuNP input (Fig. 5*B*). Subsequent cryo-EM analysis identified particles corresponding to the GluN1/GluN2A receptor bound by two 5F11 Fab-dimeric AuNPs, with an interparticle spacing of ~200 Å measured in a representative micrograph (Fig. 5*C*). Using the GluN1/2A density maps, the GluN1/2A structure (PDB: 6MMP) (31), the AlphaFold-predicted 5F11 Fab, and an AuNP template, we constructed a model of the receptor complexed with two Fab-dimeric AuNPs (Fig. 5*D*). For comparison, PEGylated 5F11 monomeric AuNPs were similarly incubated with the receptor (*SI Appendix*, Fig. S8*A*). Cryo-EM analysis revealed receptor particles decorated by two monomeric AuNPs (*SI Appendix*, Fig. S8*B*), enabling construction of the corresponding monomeric AuNP receptor model (*SI Appendix*, Fig. S8*C*). Superposition of particles from representative micrographs and their atomic models for both monomeric and dimeric AuNP complexes showed excellent agreement (*SI Appendix*, Fig. S8 *D* and *E*).

### In Situ Cryo-ET of Brain Slice Synapses Labeled With 5F11-Dimeric AuNP.

**Fig. 6.**
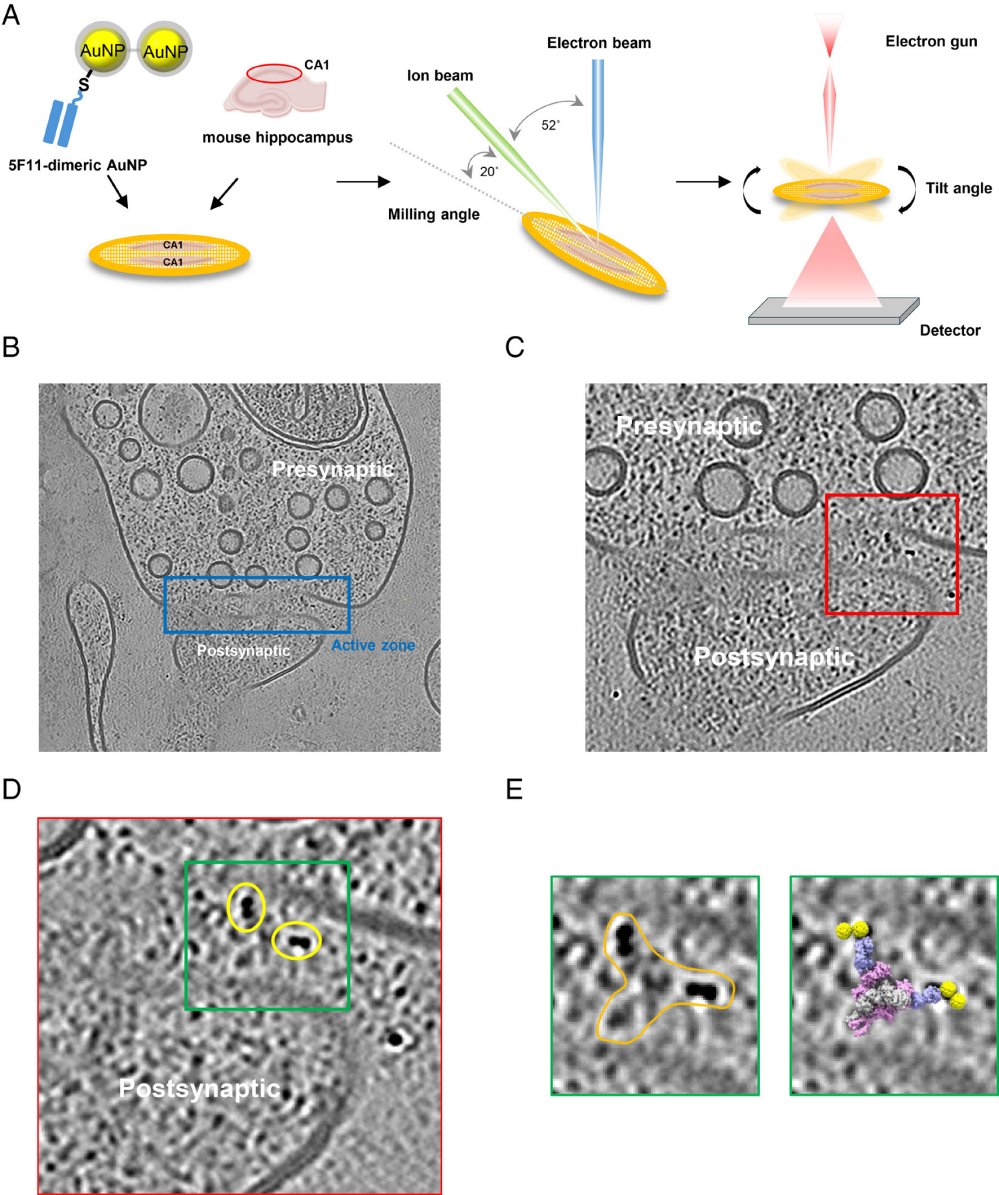
In situ cryo-ET of endogenous synapses labeled with 5F11-dimeric AuNP. (*A*) Schematic of cryo-ET workflow: 5F11-dimeric AuNP-labeled CA1 tissue is applied to grids, cryo-FIB (waffle) milled at 20°, and imaged by a −68° to +28° tilt series. (*B*) Overview of the pre- and postsynaptic structures. (*C*) Magnified view of the synaptic cleft at the active zone. (*D*) Presence of 5F11-dimeric AuNPs within the synaptic cleft (yellow oval). (*E*) The *Left* panel shows a magnified view of the green boxed region in (*D*), where two adjacent dimeric AuNPs (orange outline) are presumed to correspond to two NMDARs bound by 5F11-dimeric AuNPs. The right panel shows the same region overlaid with the modeled structure of the NMDAR-5F11-dimeric AuNP complex.

To assess the ability of the engineered 5F11 Fab-dimeric AuNP–PEG to accurately label synaptic NMDARs in situ, we extracted the hippocampal brain tissue from mice and incubated the tissue with the 5F11 Fab-dimeric AuNP–PEG conjugate using methods similar to those previously described (8). The CA1 region of hippocampus tissue containing Schaffer collaterals was manually isolated and two CA1 tissues were placed on an EM grid and then high pressure frozen in the presence of a cryoprotectant solution that included 20% dextran and 5% sucrose and Au fiducials. These hippocampus samples were milled by a focused ion beam (FIB) using the waffle method (32) with a milling angle of 20° (33). Targets for tilt series were aimed at picking the presence of synaptic features and synaptic cleft regions likely harboring NMDARs (34). Tilt series were acquired using a dose-symmetric scheme starting at −20°, normal to the lamella angle, and range between −68° to +28° with a 3° increment (Fig. 6*A*).

Three-dimensional reconstructions revealed clear visualization of both presynaptic and postsynaptic membranes (Fig. 6*B*). Within the synaptic cleft, multiple dimeric AuNPs were observed (Fig. 6*C*). Given that NMDARs contain two GluN1 subunits and are localized to the postsynaptic membrane, we identified AuNP-labeled sites that are consistent with expected NMDAR locations (Fig. 6*D*). Overlaying these data with our structural model suggests a reasonable alignment between the observed AuNPs and the predicted NMDAR positions (Fig. 6*E*). Additionally, three dimeric AuNPs were manually picked from the tomogram, and their structural features were shown in orthogonal 3D planes. In the xy-plane, the dimeric AuNPs exhibit a clearly recognizable “dumbbell” like shape, whereas in the xz- and yz-planes, the density features associated with the AuNP dimers are less well defined (*SI Appendix*, Fig. S9*A*). The “blurred” resolution in the xz- and yz-planes is likely due to a number of factors, including the “missing wedge,” and imperfections in contrast transfer function and motion correction. In addition, views of the AuNP dimers parallel, or close to parallel, to the “dumbbell” axis will reveal a single feature, rather than the clearly separated AuNPs visualized in views perpendicular to the dumbbell axis.

### Evidence for NMDAR labeling by 5F11 Fab–AuNP dimers.

To substantiate the conclusion that the 5F11 Fab–AuNP dimers label NMDARs, and to assess the impact of the Fab–AuNP conjugate on the width of the synaptic cleft, we carried out detailed measurements of the AuNP positions from the postsynaptic membrane, together with analysis of labeled and unlabeled synaptic cleft widths. Analysis of 93 dimeric AuNPs located within the synaptic cleft revealed a mean shortest distance to the postsynaptic membrane of 156 ± 28 Å (n = 93) (*SI Appendix*, Fig. S9*B*). Using the “single-particle” studies of the 5F11-AuNP and NMDAR complex as a guide, we predict minimum and maximum distances of 110 Å and 190 Å between the centers of dimeric AuNPs relative to the postsynaptic membrane. Upon analysis of the tomograms, we find that the measured mean distance of dimeric AuNPs from the postsynaptic membrane falls within this range (*SI Appendix*, Fig. S9*C*). The full tomogram used for Fig. 6 is shown in Movies S2 and S3−S9 present the seven tomograms used in *SI Appendix*, Fig. S9*B* with the manually picked dimeric AuNPs marked. The number of particles picked from each tomogram and their associated data codes are summarized in *SI Appendix*, Table S1.

To determine the extent to which Fab–AuNP dimer labeling might distort the synaptic cleft and change the spacing between pre- and postsynaptic membranes, we analyzed unlabeled synaptic tomograms from unlabeled, but otherwise identical, lamella. We not only found that the synaptic cleft revealed no particles resembling those observed in the dimeric AuNP-labeled datasets (*SI Appendix*, Fig. S10*A* and Movie S10−S13 and Table S2) but also that comparison of the mean synaptic cleft widths between dimeric AuNP-labeled and unlabeled tomograms revealed no significant difference (*SI Appendix*, Fig. S10*B*).

## Discussion

In this study, we present a strategy for constructing dimeric AuNPs conjugated to engineered Fab fragments as distinctive and biologically specific cryo-ET labels. Through a stepwise synthesis, purification, and functionalization process, we generated highly homogeneous dimeric AuNPs capable of specifically labeling NMDARs both in vitro and in situ. By integrating complementary biophysical techniques including SAXS, cryo-EM, FSEC, and tomography, we confirmed the structural integrity, labeling efficiency, and stability of the dimeric AuNP conjugates. The successful isolation of dimeric AuNPs via BPDT crosslinking and native PAGE allowed us to resolve discrete multimeric species. While monomers and dimers were readily separable, higher-order species such as trimers displayed geometric heterogeneity, making them unsuitable for uniform labeling applications. Cryo-EM and SAXS analyses consistently demonstrated that the dimeric AuNPs possess a defined monomer–monomer center-to-center distance of 13.6 Å and a maximum end-to-end extent of 56 to 58 Å, validating the reproducibility of their structural features. Engineering of the 5F11 Fab with terminal cysteines enabled site-specific conjugation to AuNPs without compromising antigen recognition, as demonstrated both by cryo-EM reconstructions showing binding to the R1 and R2 lobes of GluN1 and by FSEC monitored complex formation. Importantly, PEGylation of the Fab–AuNP conjugates was necessary to minimize nonspecific aggregation and improve stability. We note that because the AuNP monomers with the dimer are coupled by disulfide-linked BPDT molecules, the dimer may be sensitive to the reducing cytoplasmic environment. Nevertheless, because PEGylation is known to stabilize AuNPs under cellular and in vivo conditions, the PEG-surrounded BPDT–BPDT linkage may be more robust than anticipated. Further studies are required to completely address the stability of the AuNP dimers within the cytoplasmic milieu (35, 36). Among the PEG variants tested, mPEG-SH (1 kDa) conferred superior long-term stability at 4 °C, making it optimal for downstream applications.

We performed PEGylation using three different sizes of mPEG-SH: 350, 550, and 1 kDa. Notably, mPEG-SH 350 induced significantly more Fab–AuNP dissociation compared to the other PEG sizes at equivalent concentrations, as indicated by a pronounced free Fab peak (*SI Appendix*, Fig. S6*B*). While minor differences were observed, both 550 and 1 k PEGs produced similar results (*SI Appendix*, Fig. S6 *C* and *D*). However, a time-dependent stability test at 4 °C revealed that the 550 Da PEG sample, though initially stable for up to 2 d, showed progressive aggregation thereafter, with the major peak shifting toward the void volume (*SI Appendix*, Fig. S7). In contrast, the 1 kDa PEG sample remained stable even after 2 d (Fig. 3*C*). This result contrasts with our previous findings in monomeric AuNP systems, where PEG 550 was sufficient to maintain conjugate stability (8). We hypothesize that PEGylation of dimeric AuNPs poses a distinct challenge: In contrast to monomeric systems, excess PEGylation can promote the dissociation of the dimer structure. Because we specifically aim to preserve Fab-conjugated dimeric AuNPs, the final samples likely contain fewer PEG molecules per particle than in the monomeric case. This lower PEG density may reduce surface coverage and passivation, necessitating the use of larger PEGs to maintain long-term stability.

In this study, we provide evidence for the feasibility of distinguishing monomeric and dimeric AuNPs in cryotomograms. Using a machine learning algorithm, we successfully trained a classifier that discriminates between monomers and dimers, establishing a pipeline for automated, high-throughput particle annotation. This capability is critical for multiplexed labeling strategies or for resolving mixed populations in situ. When applied to recombinant NMDAR complexes, the Fab-dimeric AuNPs demonstrated robust and specific binding, with the reconstructed models matching expected geometries. Interestingly, the inter-AuNP distance (~200 Å) observed in cryo-EM matches the anticipated spacing between two Fab-bound receptor sites, reinforcing the structural fidelity of the labeling strategy (Fig. 6*E*). Also, the statistical analysis of distances between dimeric AuNPs and the postsynaptic membrane closely matches the expected position of NMDARs within the synaptic cleft, supporting the conclusion that our labeling strategy successfully targets synaptic NMDARs (*SI Appendix*, Fig. S9 *B* and *C*). Moreover, no dimeric AuNP-like particles were detected in the unlabeled clefts (*SI Appendix*, Fig. S10*A*), and comparison of mean synaptic cleft widths between labeled and unlabeled tomograms revealed no significant differences (*SI Appendix*, Fig. S10*B*), indicating that our labeling method specifically binds NMDARs without introducing any artifactual alterations to the synaptic cleft.

Most importantly, in situ cryo-ET of brain slices revealed that dimeric AuNPs labeled with 5F11 Fab can successfully target NMDARs within the postsynaptic density. These labels were spatially consistent with receptor localization and were distinguishable in 3D reconstructions. These findings highlight that dimeric AuNPs are effective tools for labeling native proteins directly in intact tissues. This capability provides a tool that may support efforts to map molecular organization in cells and neural circuits.

Notably, our system was validated in the highly confined environment of the synaptic cleft, which spans only ~28 nm between pre- and postsynaptic membranes. The ability of the dimeric AuNP labels to function effectively in such a spatially restricted compartment highlights the robustness and versatility of this labeling strategy. While the current study focused on NMDARs as a model system, the underlying design principle leveraging dual-site Fab conjugation and PEGylation can be readily adapted to study the spatial relationship between two molecular targets in situ. Given the narrow intermembrane space of the synaptic cleft, successful discrimination of dimeric versus monomeric AuNPs in this context supports the feasibility of applying this system to other cellular regions, including those with comparable or even smaller geometric constraints. Thus, this approach offers a broadly applicable tool for high-resolution molecular annotation in complex cellular environments.

While dimeric AuNPs offer enhanced labeling specificity and spatial resolution due to their defined geometry and multivalency, they also present certain limitations. First, in comparison to monomeric AuNPs, the larger size of dimeric AuNPs can increase the risk of steric hindrance when targeting densely packed or membrane-associated proteins. Second, compared to monomeric AuNPs, the synthesis and purification of dimeric AuNPs are more complex and less efficient, resulting in lower yields and requiring more time and material resources to obtain sufficient quantities of dimer. Third, high-quality tomograms are required. Because the two AuNPs in a dimeric AuNP are connected in random relative orientations, obtaining high-quality tomograms is essential to clearly distinguish dimeric AuNPs from monomeric AuNPs (*SI Appendix*, Fig. S9*A*). Additionally, in the conjugation of dimeric AuNPs with Fab fragments, typically only one of the two AuNPs engages with the Fab, while the other remains unconjugated and free to adopt a random orientation. This property may cause increased heterogeneity in the distance between the label and its target (*SI Appendix*, Fig. S9*B*). Finally, under high-density labeling conditions, when the nearest-neighbor distance between monomeric AuNPs falls below ~4.5 nm, reliable discrimination between monomeric and dimeric AuNPs becomes difficult.

In summary, our Fab-dimeric AuNP labeling strategy not only enables precise molecular tagging in highly confined environments such as the synaptic cleft but also establishes a broadly applicable framework for investigating the spatial relationships between closely positioned proteins. We anticipate that the dimeric AuNPs can be coupled to other macromolecular “binders,” such as nanobodies and darpins, thus expanding their utility. The demonstrated success in resolving monomeric and dimeric AuNPs in both purified systems and complex tissue highlights the versatility and generalizability of this method. As such, this approach holds potential for use in structural and cellular biology, particularly for studying protein complexes and nanoscale architectures in situ.

## Materials and Methods

Detailed methods are provided in *SI Appendix*. Monomeric AuNPs were synthesized using a modified Sokołowska protocol (20) with 3-MBA and HAuCl_4_ followed by NaBH_4_ reduction and methanol/water precipitation. Dimeric AuNPs were generated via BPDT-mediated ligand exchange and purified by PAGE gel extraction (18). SAXS data were collected using an Anton Paar SAXSess instrument and analyzed with IRENA/IgorPro (37). Cryo-EM data of monomer/dimer AuNPs were collected on a Krios3 microscope, processed in CryoSPARC, and classified into distinct classes. Radial profiles and center-to-center distances were computed with custom Python scripts. The 5F11 Fab targeting GluN1 was expressed in Sf9 cells, purified via Strep-Tactin resin and SEC, and conjugated to dimeric AuNPs. The conjugate was PEGylated with mPEG-SH and further purified by FSEC. Monomeric and dimeric 5F11 Fab-conjugated AuNPs, PEGylated with 1 kDa mPEG-SH were applied to glow-discharged Quantifoil gold grids and vitrified using a Vitrobot. Cryo-ET tilt series were acquired on a Titan Krios at 300 kV and reconstructed using both AreTomo (38) and IMOD (39) pipelines, with final tomograms at 10 Å pixel size. DeepETPicker (30) was then used to distinguish monomeric and dimeric AuNPs by manually seeding particle classes and iteratively refining coordinates with class-specific OCP diameters. PEGylated monomeric and dimeric AuNP-5F11 Fab conjugates were used to label recombinant GluN1/GluN2A receptors and analyzed by cryo-EM. Adult heterozygous vGlut1-mScarlet; PSD95-EGFP C57BL/6 mice were used to prepare hippocampal slices, which were incubated with PEGylated 5F11 Fab-dimeric AuNPs or untreated (unlabeled), cryofrozen, and processed by cryo-FIB milling using the “waffle” method (32) for tomogram acquisition. Dose-symmetric tilt series spanning −68° to +28° in 3° increments [starting at −20° (33)] were collected in EER (40) format: Labeled samples were acquired using SerialEM (41), while unlabeled samples were acquired using *Tomography 5* software. Cryo-ET tilt series were acquired on a Titan Krios microscope, reconstructed using AreTomo3 (42) and DeepDeWedge (43), and analyzed with MemBrain (44) and IMOD.

## Supplementary Material

Appendix 01 (PDF)

Movie S1. In-water tomogram containing a mixture of monomeric and dimeric AuNPs. The first segment presents the raw tomogram without markers, and the second applies DeepETPicker-identified markers: monomers are highlighted in red, and dimers in yellow.

Movie S2. Tomogram movie containing the pre- and postsynaptic regions shown in Figure 6.

Movie S3. Synaptic cleft tomograms (EMD-71801, EMD-71954, EMD-71955, EMD-71956, EMD-71957, EMD-71958, and EMD-71959) showing dimeric AuNPs with manually picked particles indicated by green markers. These datasets were used for distance measurements in Figure S9B.

Movie S4. Synaptic cleft tomograms (EMD-71801, EMD-71954, EMD-71955, EMD-71956, EMD-71957, EMD-71958, and EMD-71959) showing dimeric AuNPs with manually picked particles indicated by green markers. These datasets were used for distance measurements in Figure S9B.

Movie S5. Synaptic cleft tomograms (EMD-71801, EMD-71954, EMD-71955, EMD-71956, EMD-71957, EMD-71958, and EMD-71959) showing dimeric AuNPs with manually picked particles indicated by green markers. These datasets were used for distance measurements in Figure S9B.

Movie S6. Synaptic cleft tomograms (EMD-71801, EMD-71954, EMD-71955, EMD-71956, EMD-71957, EMD-71958, and EMD-71959) showing dimeric AuNPs with manually picked particles indicated by green markers. These datasets were used for distance measurements in Figure S9B.

Movie S7. Synaptic cleft tomograms (EMD-71801, EMD-71954, EMD-71955, EMD-71956, EMD-71957, EMD-71958, and EMD-71959) showing dimeric AuNPs with manually picked particles indicated by green markers. These datasets were used for distance measurements in Figure S9B.

Movie S8. Synaptic cleft tomograms (EMD-71801, EMD-71954, EMD-71955, EMD-71956, EMD-71957, EMD-71958, and EMD-71959) showing dimeric AuNPs with manually picked particles indicated by green markers. These datasets were used for distance measurements in Figure S9B.

Movie S9. Synaptic cleft tomograms (EMD-71801, EMD-71954, EMD-71955, EMD-71956, EMD-71957, EMD-71958, and EMD-71959) showing dimeric AuNPs with manually picked particles indicated by green markers. These datasets were used for distance measurements in Figure S9B.

Movie S10. Synaptic cleft tomograms (EMD-72056, EMD-72057, EMD-72058, and EMD-72059), in which no particles resembling dimeric AuNPs were detected within the cleft.

Movie S11. Synaptic cleft tomograms (EMD-72056, EMD-72057, EMD-72058, and EMD-72059), in which no particles resembling dimeric AuNPs were detected within the cleft.

Movie S12. Synaptic cleft tomograms (EMD-72056, EMD-72057, EMD-72058, and EMD-72059), in which no particles resembling dimeric AuNPs were detected within the cleft.

Movie S13. Synaptic cleft tomograms (EMD-72056, EMD-72057, EMD-72058, and EMD-72059), in which no particles resembling dimeric AuNPs were detected within the cleft.

## Data Availability

Tomograms of brain slice synapses labeled with 5F11 Fab-dimeric AuNPs have been deposited to the Electron Microscopy Data Bank (EMDB) under accession codes EMD-71801 (45), EMD-71954 (46), EMD-71955 (47), EMD-71956 (48), EMD-71957 (49), EMD-71958 (50), and EMD-71959 (51). The corresponding raw data have been deposited in the Electron Microscopy Public Image Archive (EMPIAR) under the accession code EMPIAR-12892 (52). Tomograms of unlabeled brain slice synapses have been deposited in the EMDB under accession codes EMD-72056 (53), EMD-72057 (54), EMD-72058 (55), and EMD-72059 (56), with their raw data archived in EMPIAR under accession code EMPIAR-12915 (57).
