# Supplementary material for: Dimeric gold nanoparticles enable multiplexed labeling in cryoelectron tomography
Source: Proc Natl Acad Sci U S A. 2025 Nov 24;122(48):e2524034122. doi: 10.1073/pnas.2524034122 (PMC12685141; doi:10.1073/pnas.2524034122)
Supplement: Supplementary file 1 — Appendix 01 (PDF) [file pnas.2524034122.sapp.pdf]

PNAS

Supporting Information for
Dimeric gold nanoparticles enable multiplexed labeling in cryo-electron tomography

Hoyoung Kim[1], Cathy Spangler[1], Aya Matsui[1,2], Johannes Elferich[3], Junhoe Kim[1], Alex Roseborough[4], May Nyman[4], Tanja M. Lahtinen[5], Eric Gouaux[1, 2]*

[1]Vollum Institute, Oregon Health and Science University, Portland, OR, USA

[2]Howard Hughes Medical Institute, Oregon Health and Science University, Portland, OR, USA

[3]RNA Therapeutics Institute, University of Massachusetts Chan Medical School, Howard Hughes Medical Institute, Worcester, MA, USA

[4]Department of Chemistry, Oregon State University, Corvallis, OR, 97331 USA

[5]Department of Chemistry, Nanoscience Center, University of Jyväskylä, FI-40014, Finland

*Correspondence to Eric Gouaux
Email: gouauxe@ohsu.edu

This PDF file includes:

Supporting Information Text
Figures S1 to S10
Table S1 to S2
Legends for Movies S1 to S13
SI References

Other supporting materials for this manuscript include the following:

Movies S1 to S13

Supporting Information Text

Materials and Methods

Synthesis of gold nanoparticles (AuNPs)

Synthesis of the monomeric AuNPs was based on the procedure of Sokołowska and colleagues (1). An 84 mM solution of 3-mercaptobenzoic acid (3-MBA) (Sigma) was prepared by dissolving 246 mg in 19 mL of methanol (Fisher Bioreagents). Likewise, a 33 mM solution of $HAuCl_4 \cdot 3H_2O$ (Sigma) was prepared by dissolving in 126 mg 9.7 mL of methanol. Then, 9.07 mL of the $HAuCl_4$ solution was mixed with 18.14 mL of the 3-MBA solution in a 250 mL glass beaker, followed by the addition of 68 mL of nanopure water and 5.69 mL of 2 M NaOH (Sigma) with continuous stirring until the pH reached 13. The solution was transferred to a 250 mL reaction vessel and stirred at room temperature for 20 hours. The next day, 23.21 mL of methanol and 68.28 mL of water were added, followed by 2.37 mL of freshly prepared 0.19 M $NaBH_4$ (Sigma) in water, and stirring was continued for an additional 4.5 hours. The reaction was quenched by adding 16 mL of 0.1 M NaCl (Sigma) in water and 32 mL of methanol. The mixture was divided into six pre-weight 50 mL plastic tubes and centrifuged at 3500 g for 15 minutes. Subsequently, 6 mL of a 1:3 water-methanol solution was added to each tube, pooled into a single tube, and centrifuged again at 3500 g for 15 minutes. After decanting the supernatant, the precipitates were vacuum-dried overnight using a desiccator connected to house vacuum. Finally, 1 mL of deionized water was added to dissolve the precipitates, forming an Au 3-MBA suspension, which was stored at 4°C in the dark.

Dimeric AuNP synthesis

The strategy for synthesizing the dimeric AuNPs was adapted from the methods of Lahtinen and colleagues (2). The ligand exchange reaction was carried out by first preparing a stock solution of biphenyl-4,4-dithiol (BPDT) (Sigma) by dissolving 0.015 g of BPDT in 5 mL of tetrahydrofuran (THF) (Sigma), resulting in a concentration of 13.74 mM BPDT. An aqueous solution of AuNP (MW: ~60,000 Da) at a concentration of 18.9 mg/mL (1 mL) was transferred to a 10 mL glass beaker. To this beaker, 50 µL of the BPDT stock solution (0.685 µmoles), corresponding to a 2:1 ratio of thiol groups to AuNPs, was added along with a few drops (100 µL) of 1 M NaOH. The reaction mixture was stirred for 3.5 hours at room temperature. After this period, the reaction was quenched by adding 4.1 mL of isopropanol (Fisher Bioreagents) and 206 µL of 5 M NH_4OAc (Sigma). The resulting black precipitate was then centrifuged at 3,500 g for 10 minutes at room temperature. The supernatant was carefully removed, and the pellet was washed with 100 µL of a 1:1 solution of nano pure H_2O and THF (50 µL each) to remove unreacted thiols. Following this, a second centrifugation was performed at 3,500 g for 10 minutes, after which most of the supernatant was removed using a pipette. The pellet was reprecipitated with 4.1 mL of isopropanol and 206 µL of 5 M NH_4OAc, followed by centrifugation at 3,500 g for 10 minutes. After discarding the supernatant, the pellet was dried overnight using a desiccator connected to house vacuum. Finally, the pellet was dissolved in 2 mL of H_2O and mixed with 1X TBE buffer and glycerol to achieve a final glycerol concentration of 10%. The sample was then subjected to gel electrophoresis using a 10% glycerol, 12% acrylamide/bis-acrylamide (29:1) (Bio-Rad) PAGE gel in 1X TBE buffer at 200 V for 60 minutes to separate the desired dimer from monomeric, trimeric, and other higher-ordered AuNP conjugates. The dimeric AuNP gel fragments were eluted from the gel (**Figure 1B**) and transferred to a 50 mL plastic tube containing 50 mL of 1X TBE buffer. The tube was incubated with 50 mL 1X TBE in plastic tube on a rotator at 4°C for 48 hours until the brown color was sufficiently removed from the gel fragment, indicating that most of the AuNP had left the gel and was in solution.

Small angle x-ray scattering (SAXS)

SAXS data were collected on an Anton Paar SAXSess instrument using Cu-Kα radiation (1.54 Å) and line collimation. A 2D image plate was used for data collection in the q = 0.018−2.5 $Å^{-1}$ range with the lower q range limited by the beam attenuator. Scattering data of neat water was collected for background subtraction. Sample solutions were syringe-filtered and sealed in 1.5 mm glass capillaries (Hampton Research) with paraffin wax. Scattering data was collected for 30 minutes.

SAXSQUANT was used for data collection and processing that included normalization, primary beam removal, background subtraction, desmearing, and smoothing to remove extra noise created by the desmearing routine. All analyses and curve-fitting were carried out utilizing IRENA macros with IgorPro 6.3 (Wavemetrics) software (3).

Cryo-EM sample, grid preparation, data collection and image processing for AuNPs
A sample with a defined mixture of monomeric and dimeric AuNPs was prepared at a molecular ratio of 1:2. Grids were prepared using Quantifoil R 1.2/1.3 on 300 gold mesh grids, which were glow-discharged at -15 mA for 30 seconds using a Pelco glow discharge device. Vitrification was performed using a Vitrobot Mark IV (Thermo Fisher) system under the following conditions: 3-second blotting time, 0 blotting force, over 80% humidity, and at 20°C. Single-particle cryo-EM imaging of all samples was conducted using a 300 kV Thermo Fisher Krios3 microscope equipped with a Falcon 4i camera direct electron detector at a magnification of 130kx with energy filter slit width of 6ev, -0.2um to -0.5um defocus, with a total electron dose of 50 e/Å². Data collection was carried out using SerialEM (4). A total of 4,212 cryo-EM images in EER (5) format were imported into CryoSparc, version 4.7 and motion corrected using patch motion correction followed by contrast transfer function (CTF) (6) estimation. Particles were picked by blob picking using circular blobs with 100 Å minimum and 200 Å maximum particle diameter, respectively. A total of 2,497,355 particles were picked initially. Particles were then extracted with a box size of 300 pixels and subjected to multiple rounds of 2D classification to separate monomeric and dimeric AuNPs. As a result, we obtained 79,897 monomeric AuNP particles and 245,542 dimeric AuNP particles. Monomeric AuNP particles were combined into a single 2D class, whereas dimeric AuNP particles were sorted into 20 separate 2D classes. Subsequent analyses were performed using the corresponding class_averages.mrc files.

Radial intensity profiling and center-to-center distance measurement
Monomeric and dimeric AuNP analyses were performed using custom Python scripts. First, 2D class-average of monomeric AuNPs were centered on their intensity-weighted midpoint and radial intensity profiles were computed by binning pixel values according to their integer-rounded distance from that center, symmetrizing over -50 Å to +50 Å, normalizing to 100%, and extracting cutoff distances at 50%, 20%, and 10% intensity levels to yield characteristic diameters. Second, class averages of 20 dimeric AuNP were segmented by Otsu thresholding (7) and removal of small objects to isolate each particle. The intensity-weighted centroids of the two peaks in each average were determined and connected by a straight line. Intensity values were sampled along this axis over a range of -50 Å to +50 Å around the midpoint (defined as 0 Å) and normalized to the maximum of each profile. Each normalized trace was interpolated onto a common 100-point grid, and the weighted mean profile and standard deviation (weights = number of particles per class) were calculated. The mean intensity profile (solid line) with ±1 SD shading was plotted, and the mean positions of the two peaks as well as the average center-to-center separation (±1 SD) were overlaid as markers and a double-headed arrow to illustrate the spatial relationship of the AuNP centroids. All image processing and plotting utilized NumPy (8), scikit-image (9), Pandas (10), and Matplotlib (11).

5F11 Fab expression and purification
The 5F11 Fab (12) expression construct, developed to target the GluN1 subunit of the NMDAR, was engineered analogously to the 15F1 Fab construct described by Matsui and colleagues (13). In brief, the 5F11 Fab harbors an extended heavy chain fragment containing a single hinge cysteine for AuNP conjugation, with a C-terminal sequence of KVDKKIVPRDAGAKPC, a thrombin cleavage site, and a C-terminal Twin Strep-tag. For expression, the construct was transduced into *Spodoptera frugiperda* (Sf9) insect cells using the baculovirus expression system. The infected Sf9 cells were cultured at 27°C in Sf-900 II SFM medium (Gibco) with appropriate antibiotics under constant shaking. After 72 hours of infection, cells were harvested by centrifugation at 5000 rpm for 30 minutes at 4°C, and the supernatant containing the secreted 5F11 Fab was collected and the pH was adjusted to 8.0 using Tris buffer, followed by centrifugation at 5000 RPM for 30 minutes at 4°C. The resulting supernatant was collected for further purification. Isolation of the 5F11 Fab was carried out using Strep-Tactin XT 4Flow (IBA

lifesciences) resin as follows. The collected supernatant was loaded onto a pre-equilibrated column packed with Strep-Tactin XT 4Flow resin in TBS buffer (20 mM Tris-Cl pH 8.0, 150 mM NaCl). The column was washed with 10 column volumes of the same buffer to remove non-specifically bound proteins. The bound Fab was eluted using 50 mM biotin in TBS buffer. Eluted fractions containing the 5F11 Fab were pooled and concentrated using Ultra centrifugal filters (MWCO 30 kDa) (Amicon) prior to SEC. Finally, the concentrated 5F11 Fab further purified by SEC removed aggregated protein as well as the biotin.

Conjugation of 5F11 Fab with dimeric AuNP

An aliquot consisting of 1000 µg of 5F11 Fab in 1X TBE buffer (500 µL) was incubated with 2 mM Tris(2-carboxyethyl) phosphine hydrochloride (TCEP) (Sigma) at 37°C for 1 hour. Following TCEP reduction, the reduced 5F11 Fab was purified using size exclusion chromatography and concentrated to 1 mg/mL to remove excess TCEP. The 5F11 Fab was then mixed with the dimeric AuNPs at 37°C for 30 minutes. To determine the optimal concentration ratio between Fab and dimeric AuNP before scaling up the reaction, various molar ratios ranging from 8:1 to 1:8 were tested (**Figure S5A**). As shown in Figure S5A, conjugation of Fab fragments to dimeric AuNPs yielded both monovalent (1:1) and bivalent (1:2) species. Because each AuNP dimer presents two available thiol-reactive sites, 1:2 conjugates form readily. Although a significant amount of unbound dimeric AuNP remained, the ratio that yielded the highest proportion of 1:1 conjugate was selected. After the conjugation reaction, the solution was diluted 1:1 with 1X TBE containing 10% glycerol. The entire reaction mixture was then loaded onto a 10% glycerol, 8% acrylamide/bis-acrylamide (29:1) PAGE gel and electrophoresed in 1X TBE at 200 V for 60 minutes. To extract the 5F11 Fab-dimeric AuNP conjugate, the corresponding band was excised from the gel and incubated with 50 mL of 1X TBE in a plastic tube on a rotator at 4°C for 48 hours until the brown color was sufficiently eluted from the gel fragment (**Figure S5B**).

PEGylation of 5F11 Fab-dimeric AuNP

To the 5F11 Fab-dimeric AuNP was added mPEG-SH (MW 350, 550 or 1k) (Creative PEGWorks) to a final concentration of 0.5 mM and the resulting mixture was incubated at 37°C for 60 minutes in 1X TBE. After 1 hour, to quench the reaction, the mixture was placed in an ice water bath at 4°C. Subsequently, the PEGylated 5F11 Fab-dimeric AuNP conjugate was purified using fluorescent-detection, size-exclusion chromatography (FSEC) (Superdex 200 increase) in 1X TBE. To estimate the concentration of the Fab AuNP conjugate, a 4-20% gradient SDS gel (Bio-Rad) was run alongside a known amount of the 5F11 Fab. The gel was visualized using silver staining (Pierce).

Sample preparation, data collection, and tilt-series processing of 5F11 Fab conjugated monomeric and dimeric AuNPs

Samples comprising a mixture of monomeric and dimeric AuNPs conjugated to the 5F11 Fab and PEGylated with 1 kDa mPEG-SH at a 1:2 molar ratio were applied to Quantifoil R2/1, 300-mesh gold grids that had been glow-discharged at -15 mA for 30 seconds (Pelco). Grids were vitrified in a Vitrobot Mark IV (Thermo Fisher) at 20°C and 80% humidity with a 3 second blot time and blot force setting of 0. Cryo-ET tilt series were acquired on a 300 kV FEI Titan Krios microscope equipped with a K3 direct electron detector at 42,000× magnification and a defocus of -3.5 µm. Tilt images were recorded in 3° steps from -48° to +48° using SerialEM (4). Raw movie frames were aligned in MotionCor2 (14) without dose weighting and binned to a 2.5 Å pixel size. Initial tomograms were reconstructed with fiducial-free alignment in AreTomo (15). A subset of tilt series were then processed in Etomo (IMOD) (16) using patch-tracking alignment (17), with fourfold binning during alignment and CTF correction by CTF plotter (18). Final tomograms were reconstructed at a pixel size of 10 Å.

Distinguishing monomeric and dimeric AuNPs within the tomogram

Using DeepETPicker (19) to distinguish monomeric and dimeric AuNPs within the tomogram, an initial selection of 30 particles, including monomers and dimers, was manually picked from a single tomogram. Monomers were assigned to Class 1, while dimers were designated as Class 2. Given that the tomogram voxel size was 10 Å, the OCP (Original Cryo-Particle) diameter was set

to 15 and 21, respectively. All other parameters were kept at their default settings for further processing. To improve the results, specifically to achieve more accurate classification and pick a greater number of particles, an iterative process of training, inference, and refinement of the particle coordinates was performed twice.

Expression, purification, and single-particle cryo-EM of the recombinant rat GluN1/GluN2A 5F11 Fab complex

Recombinant GluN1/GluN2A receptor construct was designed similarly to the previously described construct (20). The receptor was expressed in HEK293 cells infected at an MOI of 1:1, supplemented 10-12 hours post-infection with 10 mM sodium butyrate and channel blockers, and harvested 60 hours after infection. Cell membranes were solubilized in 1% LMNG containing buffer, clarified by ultracentrifugation, and the supernatant applied to Strep-Tactin®XT affinity resin. After elution and concentration, the receptor was polished by size-exclusion chromatography on a Superose 6 Increase column. To produce the 5F11 Fab fragment, the antibody was digested with papain at a 1:50 (w/w) papain-to-antibody ratio in the presence of 10 mM cysteine for 4 hours at 37°C. The reaction was stopped by adding 25 mM iodoacetamide to the mixture and incubating for 15 minutes at room temperature. The resulting Fab fragment was separated from the Fc fragments using Protein A/G beads, as described previously (12). The Fab was then mixed with purified GluN1/GluN2A at a 1:2.5 molar ratio, incubated on ice for 1 hour, and subjected to size-exclusion chromatography for further purification. For cryo-EM grid preparation, Quantifoil R2/1 200 gold grids were used under the same glow discharge and vitrification conditions as described above. The purified complex was concentrated to 4.6 mg/mL and added with 1 mM glycine and 1 mM glutamate before applying on grid. Single-particle cryo-EM data was collected on a 200 keV Talos Arctica equipped with a Gatan K2 camera using SerialEM, at the conditions of: a magnification of 1.142 Å/pixel, a defocus range of -1 to 2.5 µm, a dose rate of 8 e^-/pix/s, and total dose of 54 e/Å^2. Image processing and analysis were performed in CryoSPARC v3 (21). Approximately 16,400 particles were used for the reconstruction of the GluN1/GluN2A-5F11 Fab complex. For model building, the GluN1/GluN2A structure (PDB ID: 6MMP) (20) and the AlphaFold-predicted 5F11 Fab model (22) were fitted into the cryo-EM density map, shown in Figure S4.

Preparation of recombinant rat GluN1/GluN2A complexes with 5F11 Fab-monomeric or dimeric AuNPs-PEG

Purified recombinant GluN1/GluN2A receptor was incubated with 5F11 Fab-conjugated monomeric or dimeric PEGylated AuNPs at molar ratios ranging from 1:1 to 1:10. Each mixture was then subjected to size-exclusion chromatography on a Superose 6 Increase 10/300 GL column (Cytiva) equilibrated in buffer containing 20 mM Tris-Cl (pH 8.0), 150 mM NaCl, and 0.001% LMNG. The elution profile was monitored by FSEC, measuring AuNP absorbance at 500 nm and tryptophan fluorescence (Ex 280 nm, Em 335 nm) to identify the optimal receptor:AuNP ratio. Fractions corresponding to the expected complex peak were pooled and concentrated to 0.05 mg/mL for downstream analysis.

Collecting and processing cryo-EM of the GluN1/GluN2A with 5F11 Fab monomeric or dimeric AuNP

The 0.05 mg/mL recombinant rat GluN1/GluN2A receptor with 5F11 Fab monomeric or dimeric AuNP-PEG complex was supplemented with 1 mM glutamate, 1 mM glycine, 0.1 mM EDTA and 0.1 mM FOM immediately prior to grid preparation. Aliquots of 3.5 µL from each sample were applied to Quantifoil R2/1 300-mesh gold grids coated with a 2 nm continuous carbon layer that had been glow-discharged at -15 mA for 30 seconds (Pelco). Grids were vitrified in a Vitrobot Mark IV (Thermo Fisher) at 20°C and 80% humidity, using a 15 second wait time, 3 second blot time, and 0 blot force. Single particle cryo-EM data were collected on a Thermo Fisher Titan Krios G3 operating at 300 kV, equipped with a Falcon 4i direct-electron detector and a 6eV energy filter slit. Movies were recorded at 130,000× nominal magnification with a defocus range of -0.8 to -2.5 µm and a total dose of 50 e^-/Å^2. Automated acquisition was performed in SerialEM yielding EER format movies. These were imported into CryoSPARC v4.7 (21) for patch-based motion correction and contrast-transfer-function estimation.

Sample preparation of hippocampus brain tissue with 5F11 Fab dimeric AuNP-PEG or unlabeled

These steps were carried out essentially as described by Matsui et al. (2024) (13), with only minor modifications. Adult homozygous vGlut1-mScarlet and PSD95-EGFP with a C57BL/6 mice background were crossed to yield vGlut1-mScarlet (HET) / PSD95-EGFP (HET) offsprings. Six to fifteen-week-old male and female mice were anesthetized with isoflurane and decapitated, and brains were sectioned horizontally (40 µm) in HEPES buffer (150 mM NaCl, 2.5 mM KCl, 2 mM $CaCl_2$, 2 mM $MgCl_2$, 10 mM HEPES, pH 7.3) containing MK-801 (5 µM) using a Leica VT1200 vibratome. Slices were then transferred to HEPES buffer with 1 µM ZK-200775 and 1 µM RR2b throughout the rest of the experiment. Slices were incubated with 2 µg/mL (~40 nM) 5F11 Fab dimeric AuNP-PEG for 1 hour at room temperature on an orbital shaker (180 rpm) and then washed three times in HEPES buffer. CA1 regions of hippocampus were manually excised and equilibrated in HEPES containing 20% dextran for ≥30 minutes prior to high-pressure freezing (HPF). HPF of hippocampal CA1 slices was performed as described (13). Planchettes (Cu/Au, 6 mm, Type B, 0.3 mm cavity) were smoothed with sandpaper, polished with Wenol, and coated with 1-hexadecene. Extra-thick carbon, 200-mesh gold grids were glow-discharged (15 mA, 30 s) immediately before use. PEG-10 nm gold fiducials were pelleted, washed in HEPES with 20% dextran/5% sucrose, and 1.5-2 µL was applied to CA1 slices on the grid right before HPF. A bottom planchette, a middle sample grid, and a top planchette were assembled and frozen in a Leica EM ICE (~2050 bar, -196°C in <10 ms). The grids were clipped into auto grid rings for cryo-FIB/SEM. Cryo-FIB "waffle" milling was carried out on an Aquilos 2 (Thermo Fisher) following the same references. After low-mag SEM mapping (350×, 2 kV, 13 pA), grids were sputter-coated with Pt (30 mA, 15 s) and GIS-deposited organometallic Pt. Trench cuts (15 nA) and pre-cleans (7 nA) at 30° and 20° were made, followed by notch milling. Automated thinning was performed at a 20° milling angle with progressively reduced ion beam currents from 1 nA to 30 pA to yield ~150-250 nm lamellae, which were then manually polished at ±0.5° with 30 pA current.

Collected and reconstruction Cryo-ET data for dimeric AuNP labeled sample

Tilt series were acquired on a 300 keV FEI Titan Krios cryo-transmission electron microscope equipped with a spherical aberration corrector, a Gatan BioContinuum energy filter (6 eV slit width), and a Falcon 4i direct electron detector. Using SerialEM, dose-symmetric tilt series (23) were collected in EER format from -68° to +28° in 3° increments, starting at -20°. Images were recorded at a nominal magnification of 53,000× (calibrated pixel size 2.296 Å) with a total electron dose of 50 $e^-/Å^2$ per series and a target defocus of -3.5 µm. Tomogram reconstruction in Aretomo3 (24) employed an iterative motion-fitting routine (2,000 iterations, step size = 10, tolerance = 0.01), 4×4 patch-based motion correction (bin = 2; EER sampling = 2), and grouping of 2-4 tilt images per alignment step. Volumes were reconstructed at 2.296 Å pixel size with effective binning of 4.355 Å in X, Y, and Z, using 4×4 patch alignment and weighted back projection each volume was flipped and assembled into 1,600 voxel Z-stacks. For all downstream analyses, reconstructed volumes were further binned to an effective voxel size of 10 Å. The resulting tomograms were denoised using DeepDeWedge (DDW) (25). Coordinates of the postsynaptic membrane were obtained with MemBrain (26), dimeric AuNPs were manually picked in IMOD, and final renderings and movies were generated using IMOD and Fiji (27).

Collected and reconstruction cryo-ET data for unlabeled sample

Tilt series were acquired on a 300 keV Titan Krios G4 cryo-transmission electron microscope equipped with an X-FEG electron source, a SelectrisX energy filter, and a Falcon 4i direct electron detector. Using *Tomography 5* software, dose-symmetric tilt series (23) were collected in EER format from -68° to +28° in 3° increments, starting at -20°. Images were recorded at a nominal magnification of 64,000× (calibrated pixel size 1.94 Å) with a total electron dose of 50 $e^-/Å^2$ per series and a target defocus of -3.0, -3.5, and -4.0 µm. Tomogram reconstruction in Aretomo3 (24) employed an iterative motion-fitting routine (2,000 iterations, step size = 10, tolerance = 0.01), 4×4 patch-based motion correction (bin = 2; EER sampling = 2), and grouping of 2-4 tilt images per alignment step. Volumes were reconstructed at 1.94 Å pixel size with effective binning of 3.86 Å in X, Y, and Z. Using 4×4 patch alignment and weighted back

projection each volume was flipped and assembled into 1,600 voxel Z-stacks. For all downstream analyses, reconstructed volumes were further binned to an effective voxel size of 10 Å. The resulting tomograms were denoised using DDW (25). Coordinates of the postsynaptic membrane were obtained with MemBrain (26), and final renderings and movies were generated using IMOD and Fiji (27).

Extraction of active zone and average pre- and postsynaptic membrane distance calculation

From the pre- and postsynaptic membrane coordinates extracted by MemBrain (26), active zone vertices were identified by selecting all presynaptic points lying within a 40 Å Euclidean distance of the postsynaptic mesh. To limit memory usage, each mesh was down sampled to at most 100,000 vertices. The postsynaptic vertices were indexed with a SciPy cKDTree (28), and presynaptic vertices were queried in chunks of 50,000 to find all neighbors within the cutoff. Finally, the arithmetic mean of each presynaptic vertex's minimum neighbor distance (29) was computed to yield the average presynaptic-postsynaptic distance. Statistical analyses were performed on dimeric AuNP labeled ($n = 7$) and unlabeled ($n = 4$) sample sets, with group differences assessed using Welch's t-test.

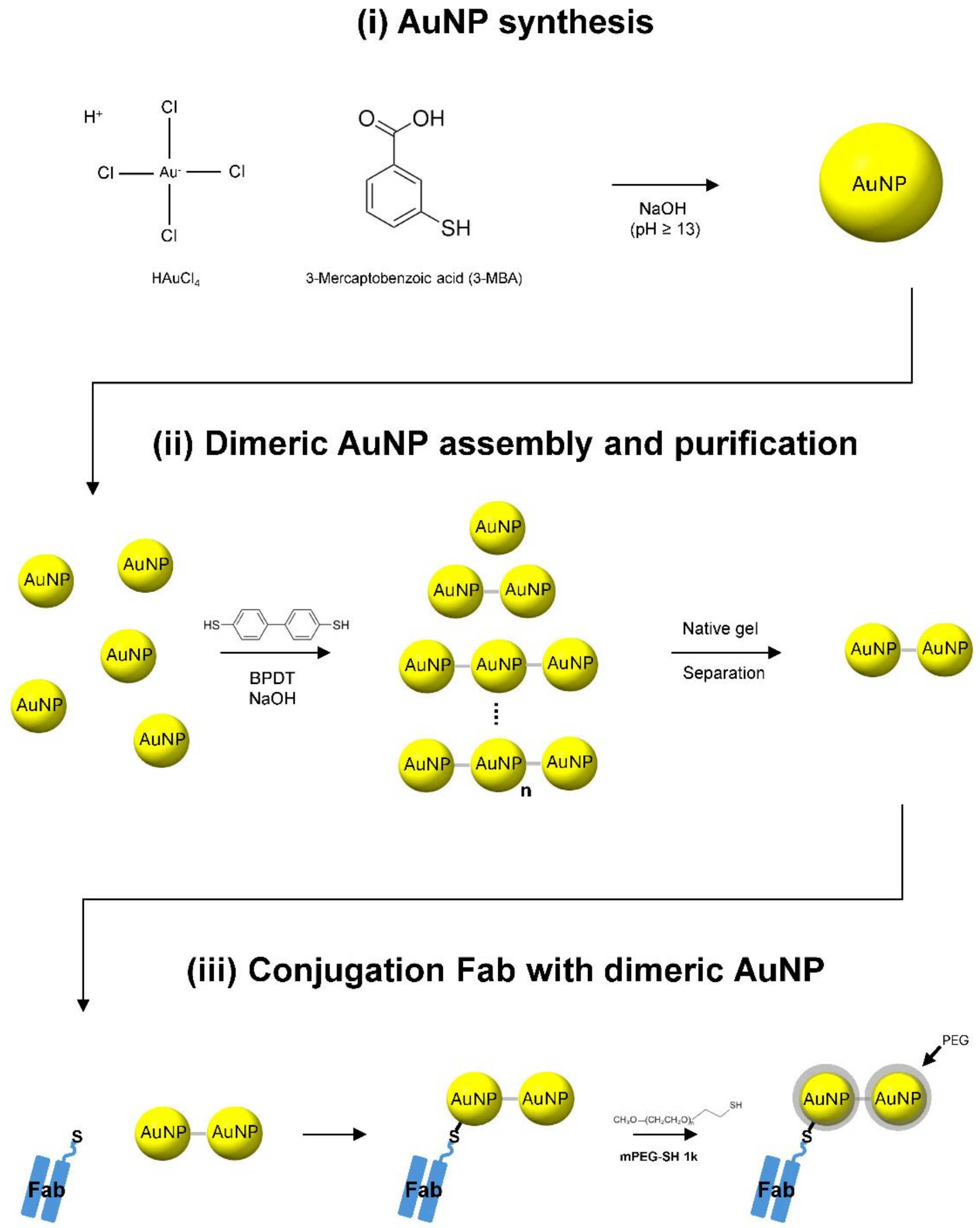

Figure S1. Schematic workflow for the synthesis, dimerization, and Fab conjugation with dimeric AuNP. This workflow has 3 main steps: (i) Monomeric AuNP synthesis. $HAuCl_4$ is reduced by 3-mercaptobenzoic acid (3-MBA) under strongly basic conditions (NaOH, pH ≥ 13), yielding 3-MBA stabilized AuNP monomers. (ii) Dimeric AuNP assembly and purification. Monomeric AuNPs are cross-linked with biphenyl-4,4′-dithiol (BPDT) in NaOH to form higher-order multimers. Dimeric AuNPs are then separated from larger assemblies by native-PAGE. (iii) Fab conjugation and PEGylation via Murray place-exchange. Purified dimeric AuNPs first undergo a Murray place-exchange reaction with thiolated antibody Fab fragments, then a second place-exchange “backfill” with 1 kDa methoxy-PEG-thiol (mPEG-SH) to impart colloidal stability and reduce nonspecific interactions.

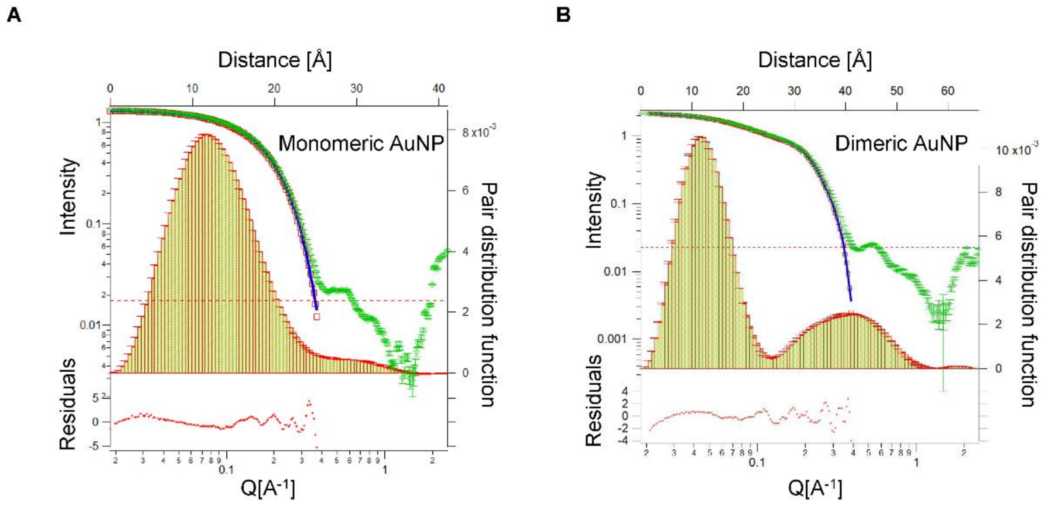

C

Parameter	Monomeric AuNP (Å)	Dimeric AuNP (Å)
**Maximum extent D_{max}**	40	65
**Basis functions used**	4	7
**Scale factor**	1.3076 ± 0.00021	2.2105 ± 0.00101
**Radius of gyration R_g**	9.9632 ± 0.00817	16.499 ± 0.09962
**Background**	0.009938 ± 0.000250	0.0041853 ± 0.000179
**Reduced X^2**	1.5255	1.0206

Figure S2. PDDF fitting of the SAXS curves using the method of (**A**) monomeric AuNP and (**B**) dimeric AuNP. The green data points represent the original scattering data (bottom axis). The red squares indicate the fitted region of the curve, after subtracting a flat background (horizontal red dashed line). The green and red bar graph represents the probability distribution of scattering vectors (top axis). The blue solid line represents the fitted curve. (**C**) Summary table of PDDF fitting results for monomeric and dimeric AuNPs, listing maximum extent (D_{max}), number of basic functions used, scale factor, radius of gyration (R_g), background level, and reduced X^2 for direct comparison.

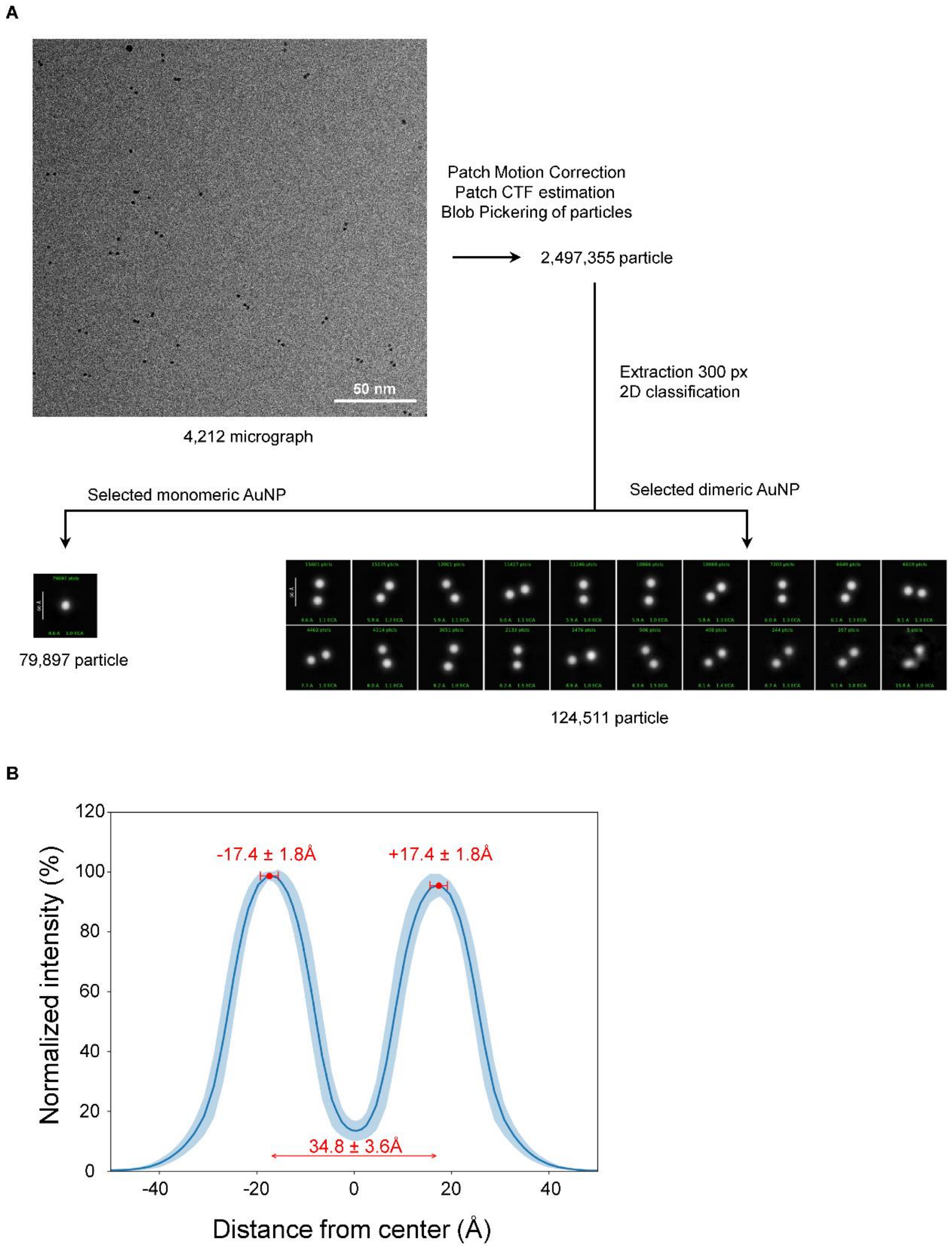

Figure S3. Cryo-EM image processing and radial profile analysis of AuNP class averages. (**A**) Workflow schematic: 4,212 raw micrographs were subjected to patch motion correction, CTF estimation, and blob picking to yield 2,497,355 particles. Particles were extracted (300px) and 2D classified into monomeric (79,897 particles) and dimeric (124,511 particles) AuNP groups. (**B**) Weighted average of normalized intensity profiles sampled along the axis connecting the centroids of each dimeric AuNP in 20 class averages. Individual profiles were measured from -50 Å to +50 Å around the midpoint of the two centroids and normalized to their own maxima. The

thin solid blue line shows the weighted mean profile (weights = number of particles per class), and the light blue, thick shaded line represents the weighted standard deviation (±SD) across classes. Red circles mark the mean positions of the two peaks (mean ± SD), and the red double-headed arrow indicates the average center-to-center separation (mean ± SD).

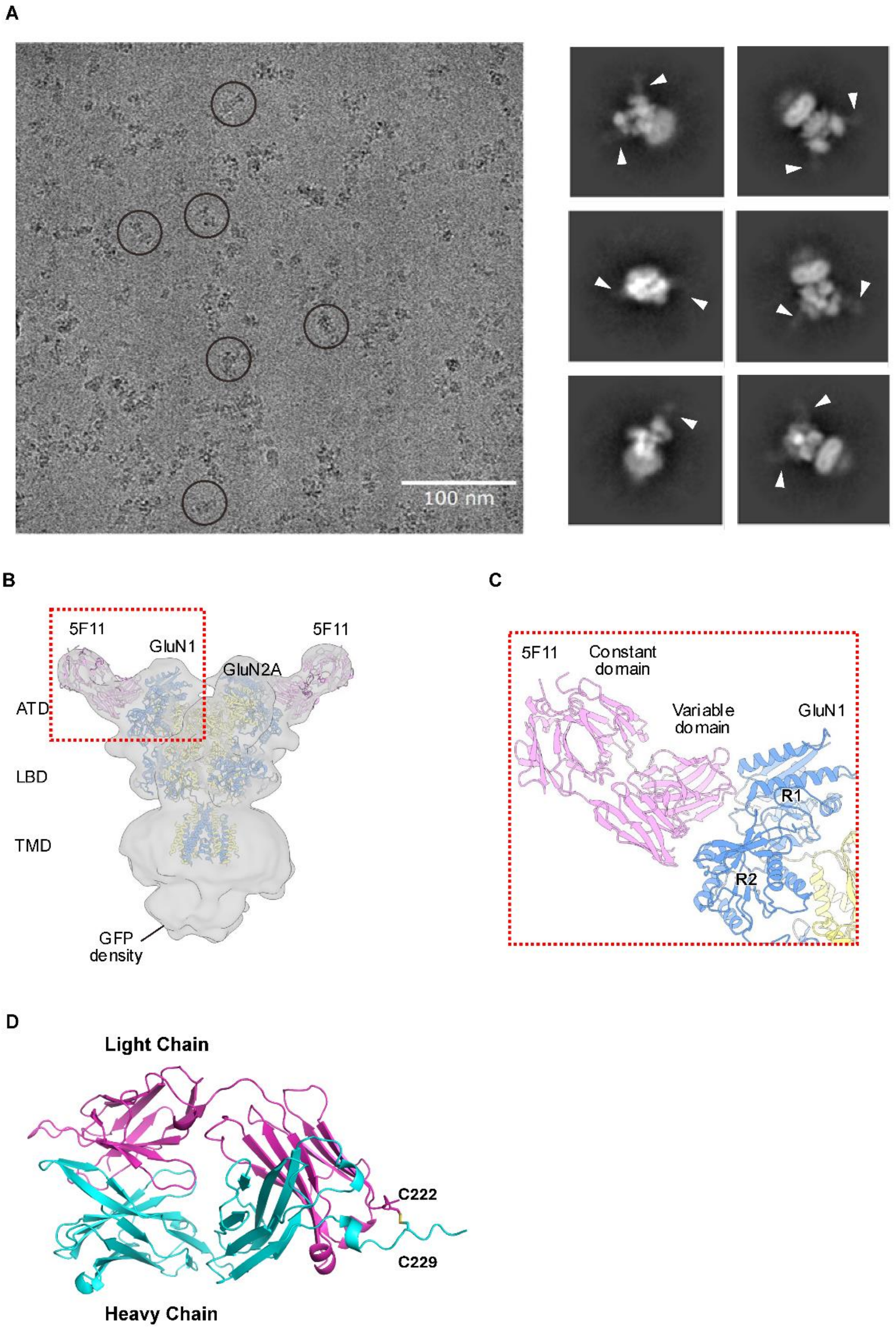
A
100 nm
B
5F11
GluN1
GluN2A
5F11
ATD
LBD
TMD
GFP
density
C
5F11
Constant
domain
Variable
domain
GluN1
R1
R2
D
Light Chain
C222
C229
Heavy Chain

Figure S4. Imaging the GluN1/GluN2A-5F11 Fab complex by single particle cryo-EM. (**A**) Representative cryo-EM micrograph showing GluN1/GluN2A-5F11 Fab complex particles (black circles). 2D class averages reveal distinct density corresponding to receptor-bound 5F11 Fab (white arrows). (**B**) 3D reconstruction of the complex with the fitted atomic model of GluN1/GluN2A-5F11 Fab. (**C**) Zoomed-in view (red dashed box) highlighting the interaction interface between the R1 and R2 domains of GluN1 and the bound 5F11 Fab. (**D**) AlphaFold3 model of the AuNP conjugatable 5F11 Fab. Following reduction, the cysteine residues C229 (heavy chain) and C222 (light chain) are predicted to be solvent accessible and suitable for AuNP conjugation.

A

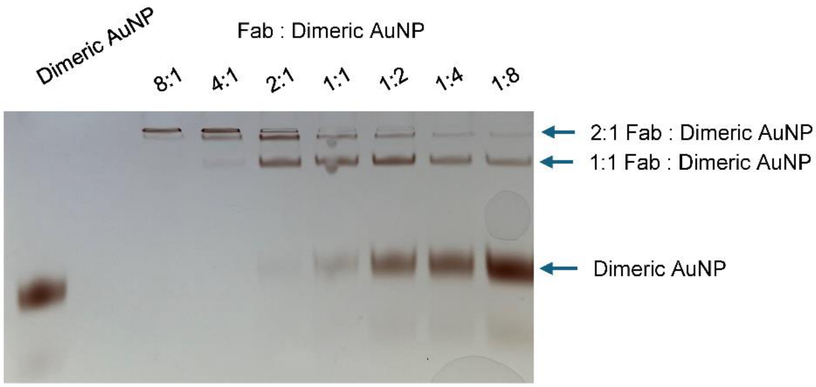

B

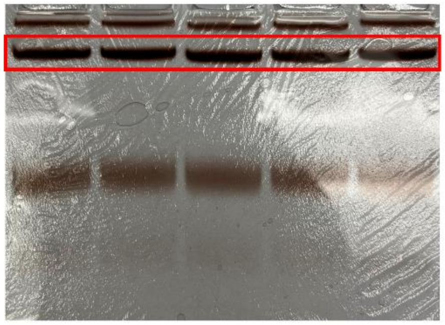

Figure S5. Conjugation and elution of 5F11 Fab with dimeric AuNP. (**A**) Screening of the molar ratio between Fab and dimeric AuNPs by native gel electrophoresis to determine the optimal ratio. (**B**) Large-scale conjugation of Fab with dimeric AuNP using the optimized ratio. The red box indicates the band corresponding to a 1:1 Fab:dimeric AuNP conjugate.

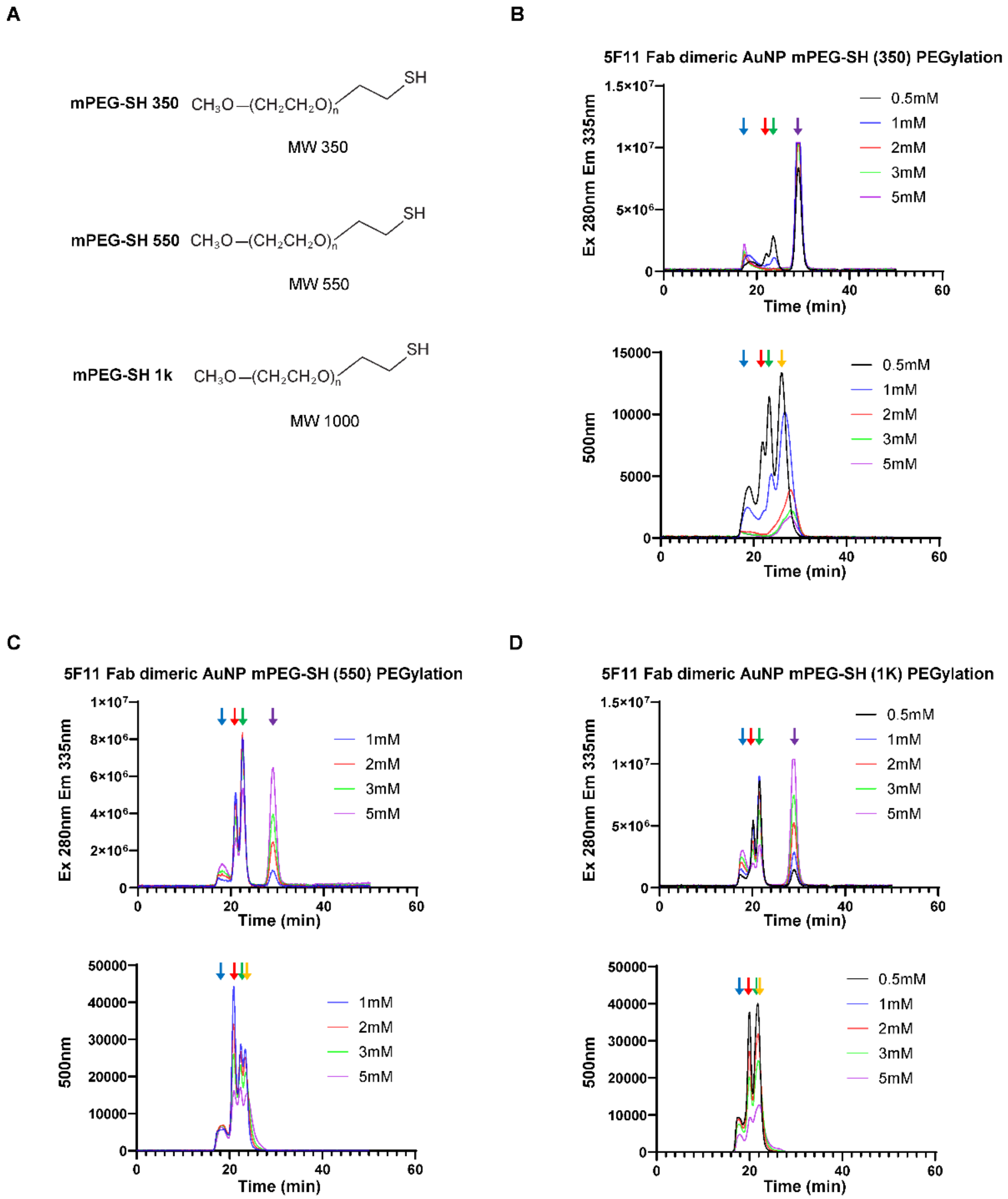

Figure S6. Determination of optimal conditions for PEGylation of the 5F11 Fab dimeric AuNP. (**A**) Chemical structures of mPEG-SH with molecular weights of 350, 550, and 1K. (**B-D**) PEGylation tests of 5F11 Fab dimeric AuNP with mPEG-SH at varying concentrations (0.5 mM - 5 mM): (B) mPEG-SH 350, (C) mPEG-SH 550, and (D) mPEG-SH 1K. In panels B-D, colored arrows denote the following: blue, aggregation peak; red, PEGylated 5F11 Fab-dimeric AuNP; green, PEGylated 5F11 Fab-monomeric AuNP; orange, PEGylated monomeric AuNP; and purple, free 5F11 Fab.

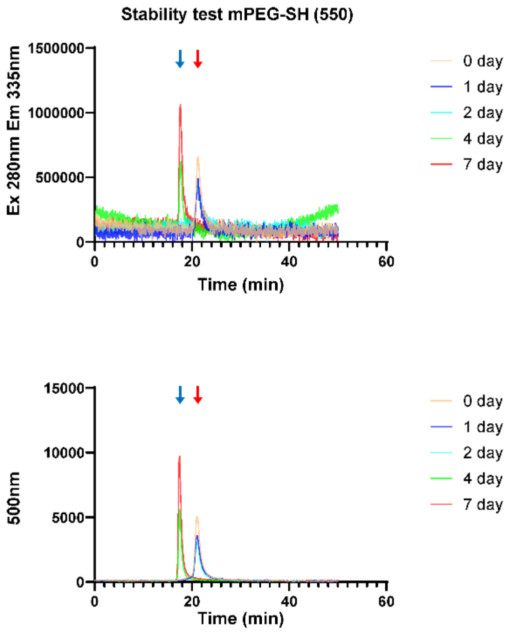

Figure S7. PEGylation and stability analysis of 5F11 Fab-dimeric AuNP-mPEG-SH (550). FSEC-based stability test of 5F11 Fab-dimeric AuNP-mPEG-SH (550) over time (day 0 to day 7) at 4°C. Colored arrows indicate the following: blue, aggregation peak; red, PEGylated 5F11 Fab-dimeric AuNP. The mPEG-SH (550)-PEGylated sample remained stable for the first 2 days, but by Day 4, the main peak shifted toward the aggregation peak, indicating a loss of stability.

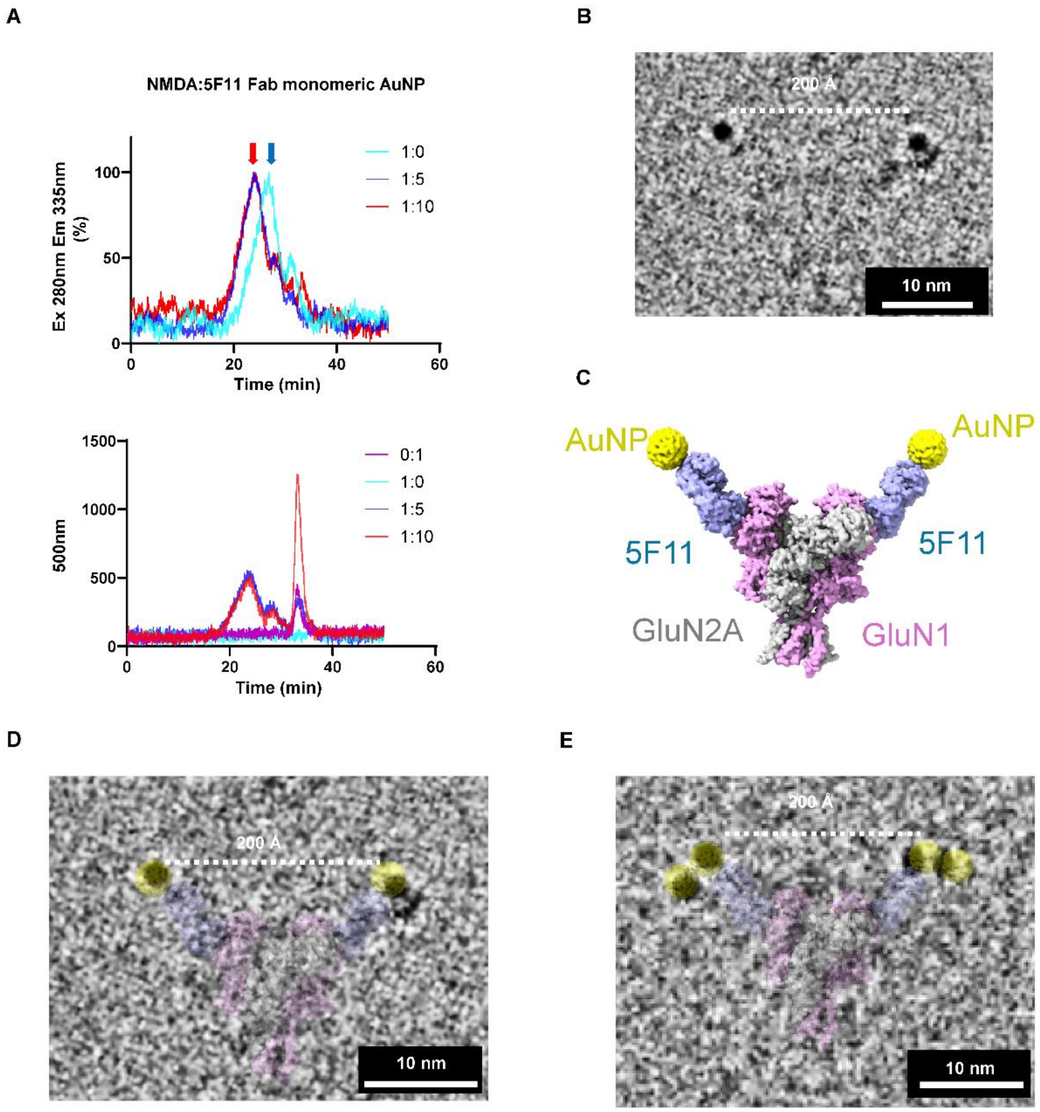

Figure S8. Binding of 5F11 Fab-monomeric AuNP to the NMDAR. (**A**) FSEC analysis of recombinant GluN1/GluN2A mixed with PEGylated 5F11-monomeric AuNP at molar ratios of 1:0, 1:5, and 1:10 (GluN1/2A: PEG-5F11-monomeric AuNP). Tryptophan fluorescence (Ex 280 nm, Em 335 nm) exhibits a clear peak shift upon complex formation (red arrow: complex; blue arrow: unbound receptor), and the AuNP absorbance at 500 nm shows an increased peak height at the same elution volume. (**B**) Representative cryo-EM micrographs confirming binding of AuNP monomers to the GluN1/GluN2A receptor. (**C**) Structural model of the GluN1/GluN2A 5F11-monomeric AuNP complex. Overlays of the cryo-EM density maps with the fitted atomic models of monomeric (**D**) and dimeric (**E**) AuNPs.

A

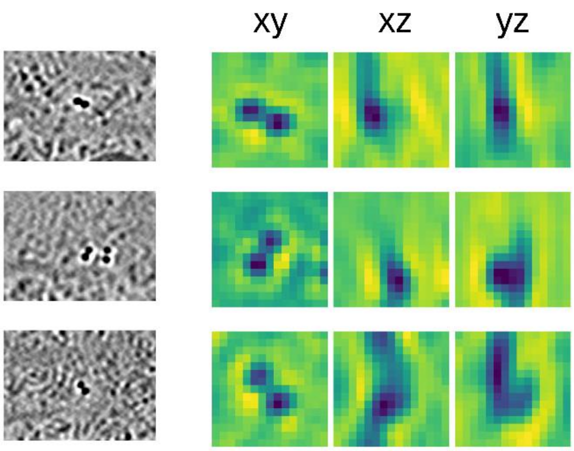

B

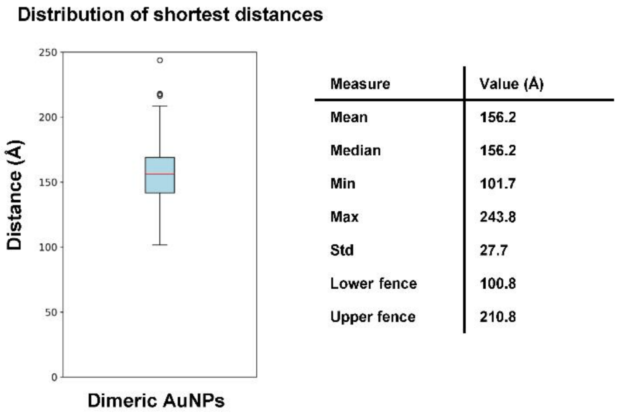

Measure	Value (Å)
Mean	156.2
Median	156.2
Min	101.7
Max	243.8
Std	27.7
Lower fence	100.8
Upper fence	210.8

C

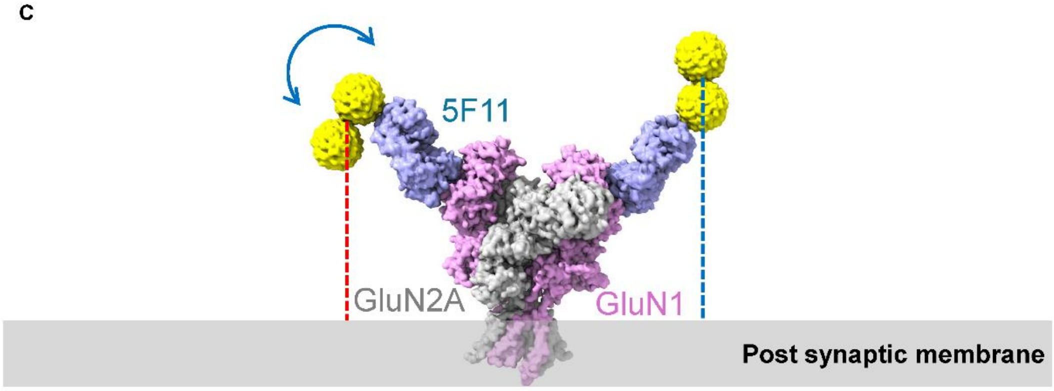

Figure S9. Three representatives dimeric AuNPs located within the synaptic cleft and quantified distance from the postsynaptic membrane. (**A**) Coordinates for each dimer were manually picked in 3dmod (IMOD): a model point was placed at the particle center in the high-contrast XY slice to define xy, then the Z-stack was scrolled to record z. The resulting (x, y, z) values were used to extract 150 × 150 Å orthogonal views: (left) XY at the dimer's Z, (center) XZ at its Y, and (right) YZ at its X (the far-left slice corresponds to the tomogram's XY plane). (**B**) The dimeric AuNPs located within the synaptic cleft were manually picked for distance measurement. The shortest distances from the postsynaptic membrane to each dimeric AuNP are quantified across these 93 particles. The boxplot summarizes the distribution of these distances. (**C**) Schematic illustrating the source of measurement variability for dimeric AuNP labels. Because only one of the two AuNPs is conjugated with Fab and the other remains free to adopt random orientations, using the geometric center of the dimer to calculate its distance from the postsynaptic membrane results in a deviation. In this diagram, the red dashed line indicates the expected minimum distance, and the blue dashed line indicates the expected maximum distance.

A

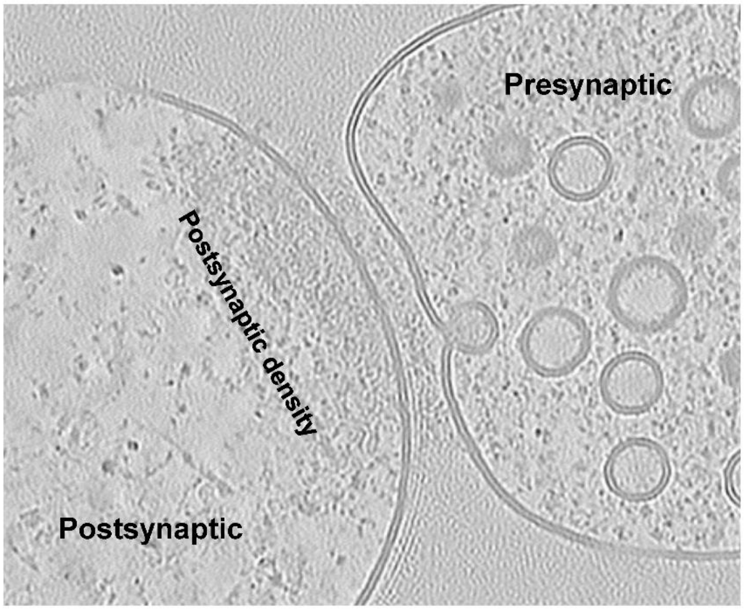
Presynaptic
Postsynaptic density
Postsynaptic

B

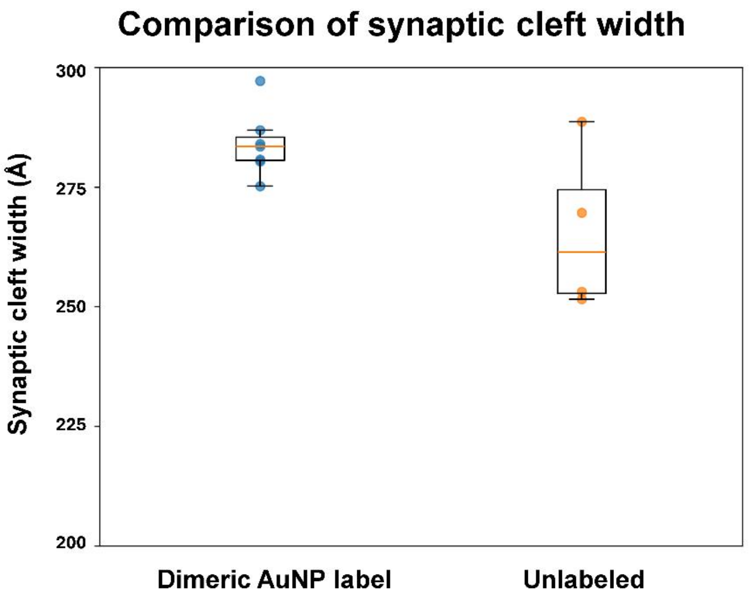
Comparison of synaptic cleft width
Synaptic cleft width (Å)
300
275
250
225
200
Dimeric AuNP label
Unlabeled

Figure S10. Comparison of synaptic cleft morphology in unlabeled tomograms and mean cleft widths between dimeric AuNP labeled and unlabeled samples. (**A**) In the unlabeled tomograms, two adjacent tissue segments are visible. One segment exhibits postsynaptic density, while the other is rich in presynaptic vesicles. These segments correspond to the post- and presynaptic regions, respectively. Unlike the datasets labeled with dimeric AuNPs, these unlabeled tomograms contain no dimeric particles within the synaptic cleft. (**B**) Bar graph comparing the average synaptic cleft widths between dimeric AuNP labeled and unlabeled samples. Labeled tomograms exhibited a mean cleft width of 284 ± 6.9 Å ($n = 7$), whereas the unlabeled tomograms showed a mean width of 266 ± 17.4 Å ($n = 4$). Statistical analysis by Welch's t-test gave a P value of 0.123. The voxel size of the tomogram is 10 Å.

Table S1. Summary of datasets: number of dimeric AuNP particles per tomogram (used in Figure S9B) and associated movies.

EMDB Code	Dimeric AuNP Count	Movies (Supplementary)
EMD-71801	12	Movie S2, Movie S3
EMD-71954	12	Movie S4
EMD-71955	5	Movie S5
EMD-71956	14	Movie S6
EMD-71957	21	Movie S7
EMD-71958	7	Movie S8
EMD-71959	22	Movie S9

Table S2. EMDB accession codes and corresponding Tomogram movies for all unlabeled Tomogram datasets.

EMDB Code	Movies (Supplementary)
EMD-72056	Movie S10
EMD-72057	Movie S11
EMD-72058	Movie S12
EMD-72059	Movie S13

Movie S1 (separate file). In-water tomogram containing a mixture of monomeric and dimeric AuNPs. The first segment presents the raw tomogram without markers, and the second applies DeepETPicker-identified markers: monomers are highlighted in red, and dimers in yellow.

Movie S2 (separate file). Tomogram movie containing the pre- and postsynaptic regions shown in Figure 6.

Movie S3-S9 (separate files). Synaptic cleft tomograms (EMD-71801, EMD-71954, EMD-71955, EMD-71956, EMD-71957, EMD-71958, and EMD-71959) showing dimeric AuNPs with manually picked particles indicated by green markers. These datasets were used for distance measurements in Figure S9B.

Movie S10-S13 (separate files). Synaptic cleft tomograms (EMD-72056, EMD-72057, EMD-72058, and EMD-72059), in which no particles resembling dimeric AuNPs were detected within the cleft.

SI References

1. K. Sokołowska *et al.*, Towards Controlled Synthesis of Water-Soluble Gold Nanoclusters: Synthesis and Analysis. *The Journal of Physical Chemistry C* **123**, 2602-2612 (2019).
2. T. Lahtinen *et al.*, Covalently linked multimers of gold nanoclusters Au102(p-MBA)44 and Au~250(p-MBA)n. *Nanoscale* **8**, 18665-18674 (2016).
3. J. Ilavsky, P. Jemian, Irena: Tool Suite for Modeling and Analysis of Small Angle Scattering. *Journal of Applied Crystallography - J APPL CRYST* **42**, 347-353 (2009).
4. D. N. Mastronarde, Automated electron microscope tomography using robust prediction of specimen movements. *Journal of Structural Biology* **152**, 36-51 (2005).
5. H. Guo *et al.*, Electron-event representation data enable efficient cryoEM file storage with full preservation of spatial and temporal resolution. *IUCrJ* **7**, 860-869 (2020).
6. A. Rohou, N. Grigorieff, CTFFIND4: Fast and accurate defocus estimation from electron micrographs. *Journal of Structural Biology* **192**, 216-221 (2015).
7. N. Otsu, A Threshold Selection Method from Gray-Level Histograms. *IEEE Transactions on Systems, Man, and Cybernetics* **9**, 62-66 (1979).
8. C. R. Harris *et al.*, Array programming with NumPy. *Nature* **585**, 357-362 (2020).
9. S. van der Walt *et al.*, scikit-image: image processing in Python. *PeerJ* **2**, e453 (2014).
10. W. McKinney, *Data Structures for Statistical Computing in Python* (2010), 10.25080/Majora-92bf1922-00a, pp. 56-61.
11. J. Hunter, Matplotlib: A 2D Graphics Environment. *Computing in Science & Engineering* **9**, 90-95 (2007).
12. N. Sheldon *et al.*, Generation of Conformation-Specific Monoclonal Antibodies for Integral Membrane Proteins. *Curr Protoc* **5**, e70142 (2025).
13. A. Matsui *et al.*, Cryo-electron tomographic investigation of native hippocampal glutamatergic synapses. *eLife* **13**, RP98458 (2024).
14. S. Q. Zheng *et al.*, MotionCor2: anisotropic correction of beam-induced motion for improved cryo-electron microscopy. *Nature Methods* **14**, 331-332 (2017).
15. S. Zheng *et al.*, AreTomo: An integrated software package for automated marker-free, motion-corrected cryo-electron tomographic alignment and reconstruction. *Journal of Structural Biology: X* **6**, 100068 (2022).
16. J. R. Kremer, D. N. Mastronarde, J. R. McIntosh, Computer Visualization of Three-Dimensional Image Data Using IMOD. *Journal of Structural Biology* **116**, 71-76 (1996).
17. D. N. Mastronarde, S. R. Held, Automated tilt series alignment and tomographic reconstruction in IMOD. *Journal of Structural Biology* **197**, 102-113 (2017).
18. D. N. Mastronarde, Accurate, automatic determination of astigmatism and phase with Ctfplotter in IMOD. *Journal of Structural Biology* **216**, 108057 (2024).
19. G. Liu *et al.*, DeepETPicker: Fast and accurate 3D particle picking for cryo-electron tomography using weakly supervised deep learning. *Nature Communications* **15**, 2090 (2024).
20. F. Jalali-Yazdi, S. Chowdhury, C. Yoshioka, E. Gouaux, Mechanisms for Zinc and Proton Inhibition of the GluN1/GluN2A NMDA Receptor. *Cell* **175**, 1520-1532.e1515 (2018).
21. A. Punjani, J. L. Rubinstein, D. J. Fleet, M. A. Brubaker, cryoSPARC: algorithms for rapid unsupervised cryo-EM structure determination. *Nature Methods* **14**, 290-296 (2017).
22. J. Jumper *et al.*, Highly accurate protein structure prediction with AlphaFold. *Nature* **596**, 583-589 (2021).
23. W. J. H. Hagen, W. Wan, J. A. G. Briggs, Implementation of a cryo-electron tomography tilt-scheme optimized for high resolution subtomogram averaging. *Journal of Structural Biology* **197**, 191-198 (2017).
24. A. Peck *et al.*, AreTomoLive: Automated reconstruction of comprehensively-corrected and denoised cryo-electron tomograms in real-time and at high throughput. *bioRxiv* 10.1101/2025.03.11.642690, 2025.2003.2011.642690 (2025).
25. S. Wiedemann, R. Heckel, A deep learning method for simultaneous denoising and missing wedge reconstruction in cryogenic electron tomography. *Nature Communications* **15**, 8255 (2024).

26. L. Lamm *et al.*, MemBrain: A deep learning-aided pipeline for detection of membrane proteins in Cryo-electron tomograms. *Computer Methods and Programs in Biomedicine* **224**, 106990 (2022).
27. J. Schindelin *et al.*, Fiji: an open-source platform for biological-image analysis. *Nature Methods* **9**, 676-682 (2012).
28. P. Virtanen *et al.*, SciPy 1.0: fundamental algorithms for scientific computing in Python. *Nature Methods* **17**, 261-272 (2020).
29. J. L. Bentley, Multidimensional binary search trees used for associative searching. *Commun. ACM* **18**, 509–517 (1975).
